# SINC-seq: correlation of transient gene expressions between nucleus and cytoplasm reflects single-cell physiology

**DOI:** 10.1186/s13059-018-1446-9

**Published:** 2018-06-06

**Authors:** Mahmoud N. Abdelmoez, Kei Iida, Yusuke Oguchi, Hidekazu Nishikii, Ryuji Yokokawa, Hidetoshi Kotera, Sotaro Uemura, Juan G. Santiago, Hirofumi Shintaku

**Affiliations:** 1 Department of Micro Engineering, Graduate School of Engineering, Kyoto University, Kyoto, Japan; 2 Microfluidics RIKEN Hakubi Research Team, RIKEN Cluster for Pioneering Research, Saitama, Japan; 3 Medical Research Support Center, Graduate School of Medicine, Kyoto University, Kyoto, Japan; 4 Department of Biological Sciences, Graduate School of Science, The University of Tokyo, Tokyo, Japan; 5 Department of Hematology, Faculty of Medicine, University of Tsukuba, Tsukuba, Japan; 6 Department of Mechanical Engineering, Stanford University, Stanford, CA USA

**Keywords:** Single cell, RNA-seq, Microfluidics, RNA transport, Splicing, Isotachophoresis, Nucleus, Cytoplasm

## Abstract

We report a microfluidic system that physically separates nuclear RNA (nucRNA) and cytoplasmic RNA (cytRNA) from a single cell and enables single-cell integrated nucRNA and cytRNA-sequencing (SINC-seq). SINC-seq constructs two individual RNA-seq libraries, nucRNA and cytRNA, per cell, quantifies gene expression in the subcellular compartments, and combines them to create novel single-cell RNA-seq data. Leveraging SINC-seq, we discover distinct natures of correlation among cytRNA and nucRNA that reflect the transient physiological state of single cells. These data provide unique insights into the regulatory network of messenger RNA from the nucleus toward the cytoplasm at the single-cell level.

**Electronic supplementary material:**

The online version of this article (10.1186/s13059-018-1446-9) contains supplementary material, which is available to authorized users.

## Background

Single-cell sequencing is a powerful tool for exploring epigenetic, genomic, and transcriptional heterogeneities at an unprecedented resolution [1–6]. RNA-seq with single nuclei (nucRNA-seq) [7, 8] is an emerging option for profiling gene expressions of cells in tissues that cannot be readily dissociated, such as the adult brain and frozen samples. nucRNA-seq is further capable of coupling with sorting by fluorescence-activated cell sorters [4, 7–9], Fluidigm C1 [5], and Drop-seq [10], and has demonstrated feasibilities of identifying cell types and cell cycles [11] with nucRNA-seq data. Grindberg et al. [7] reported that the gene expression with single nuclei is similar to that with entire single cells, with only 3.5% of the genes exhibiting differential expression. However, this was performed by comparing nucRNA-seq vs. single-cell RNA-seq (scRNA-seq) from different single cells. Such studies hypothesize that the nucRNA expression is representative of whole cells, but, to date, there has been no direct evidence of the nuclear-to-cytoplasmic correlation. Investigating the cytoplasmic RNA (cytRNA) and nuclear RNA (nucRNA) expressions with the same single cell are essential to assess the validity of using nucRNA-seq, especially for the analysis of transient biological processes.

Recent technical advances have enabled combined sequencing at multi-omic levels within the same single cells [12–14] and helped us to understand the links underlying the regulatory cascade. Several microfluidic [15–18] and non-microfluidic protocols [19, 20] offer parallel transcriptional and genomic analyses on the same single cell by fractionating the cytRNAs and nuclei of single cells. However, we know of no work that has reported an integrated nucRNA-seq and cytRNA-seq with the same cell to study RNA transport and gene regulation and function through splicing of precursor messenger RNA (pre-mRNA) [21, 22].

Here we demonstrate a novel single-cell sequencing method, SINC-seq, which combines a microfluidic protocol that physically fractionates nuclear and cytoplasmic RNAs and a subcellular RNA-seq pipeline to dissect RNA expressions in the individual subcellular compartment. We use SINC-seq to explore both correlated and uncorrelated gene expression between the compartments of single K562 human leukemic cells. We further explore correlation dynamics that reflect the transient response of differentiating K562 cells vs. erythroid cells under a perturbation of sodium butyrate (NaB), a histone deacetylase inhibitor. We show that the epigenetic modification via NaB changes the degree of correlation between cytRNA and nucRNA expression substantially. These data reveal how eukaryotes manage subcellular RNA expressions via intercompartment regulation.

## Results

### A microfluidic platform for single-cell integrated nuclear and cytoplasmic RNA-sequencing: SINC-seq

**Fig. 1:**
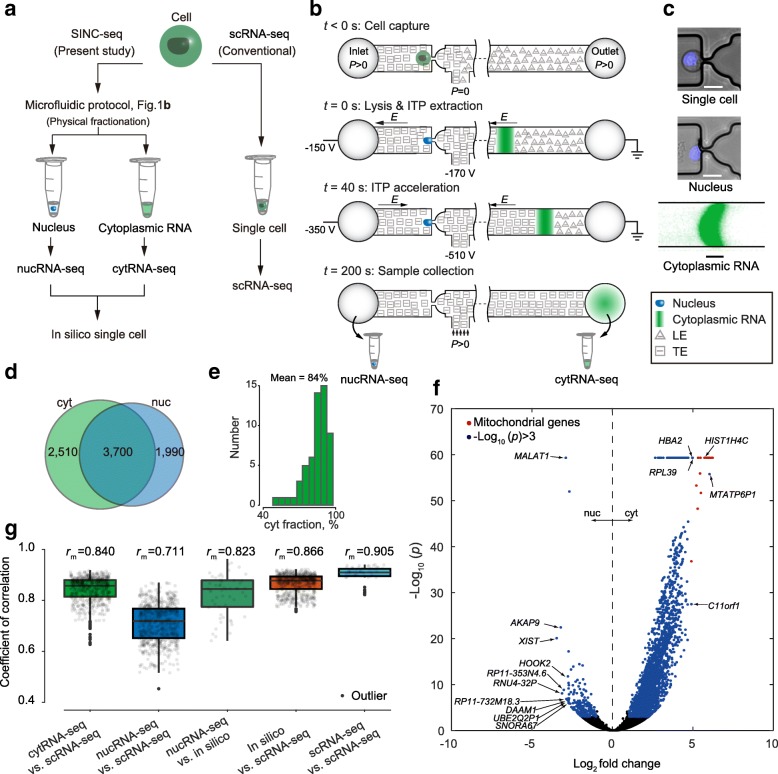
Single-cell integrated nuclear and cytoplasmic RNA-seq (*SINC-seq*). **a** SINC-seq and conventional scRNA-seq. **b** Workflow of SINC-seq. Single-cell isolation at a hydrodynamic trap via pressure-driven flow (*t* = 0 s); lysis of cell membrane and cytRNA extraction with isotachophoresis (*ITP*)-aided nucleic acid extraction (*t* > 0 s); ITP acceleration by changing voltages (*t* = 40 s); voltage deactivation and sample collection from the wells of the microchannel (*t* > 200 s). **c** Fluorescence microscopy images of the trapped single cell and nucleus after cytRNA extraction (stained with Hoechst) and extracted cytRNA stained with SYBR Green II. Scale bars are 20 μm. **d** Venn diagram of mean numbers of detected genes in cytRNA-seq and nucRNA-seq. **e** Percent proportion of abundance of transcripts in the cytoplasm. **f** Differential expression analysis between cytRNA and nucRNA. Genes enriched in cytRNA are on the *right-hand side*. *Blue*, genes with *p* values less than 0.001 and absolute log2 fold changes greater than unity. **g** Correlation coefficients of gene expression pattern computed with respect to the conventional scRNA-seq; our novel in silico single-cell normalization showed the best correlation with the scRNA-seq. We also include correlation of nucRNA vs. its in silico single cell


To dissect transcriptional correlation in the subcellular compartments, we designed SINC-seq to combine electrophoretic fractionation of cytRNA from the nucleus [16–18] with off-chip RNA sequencing (Fig. 1a, b). SINC-seq constructs individual RNA-seq libraries with cytRNA and nucRNA and integrates the sequencing data in a new format which we term an “in silico single cell.” SINC-seq starts with a microfluidic protocol that leverages a hydrodynamic trap that captures a single cell and then concentrates an electric field to selectively lyse the cytoplasmic membrane while leaving the nuclear membrane relatively intact. The trap also retains the cell nucleus during an electric field-based extraction of cytRNA that is initiated within 1 s of the electric field activation (see Fig. 1b, c, Additional file 1: Figure S1, Additional file 2: Movie S1, and Methods). Hence, our microfluidic protocol enables subcellular fractionation by coupling electric lysis [23, 24] and rapid transition to isotachophoresis (ITP)-based nucleic acid extraction from single cells [16, 17]. We confirmed that the nuclei retained their integrity by, for example, staining genomic DNA with Hoechst (Fig. 1c). The microfluidic system completes the entire process with voltage control via three end-channel electrodes and outputs the cytRNA and nucleus to different wells in less than 5 min. We note that the hydrodynamic trap integrated in this work couples hydrodynamic flow and electric field concentration and enables approximately 20-fold reduction in the applied voltage compared to our previous protocol [16–18]. Further, the microfluidic design allows a buffer exchange for the single cell using pressure-driven flow and introduction of the ITP buffer chemistry. This approach helps reduce the background noise associated with cell-free RNA in the original cell solution (Additional file 1: Figure S1e). These key improvements allowed us to study RNA expressions in subcellular compartments of single cells systematically. We serially processed single cells and prepared about eight pairs of RNA-seq libraries per day. We hope to automate (and parallelize) the SINC-seq protocol in the future and thereby increase the rate of single-cell processing.

**Additional file 2:** **Movie S1.** Electrical lysis and RNA extraction visualized by SYBR Green II. (MOV 1279 kb)


We note that subcellular fractionation of proteins from single cells by electroporation was first reported by Lu and co-workers [23, 24]. Our method leverages a similar subcellular fractionation via electric field and also uniquely enables RNA sequencing by delivering the subcellular components to two independent downstream extraction ports, including the cytRNA fraction transported via ITP [16, 17]. We hope to further extend our protocol and perhaps enable protein analyses in the future (see Qu et al. [25] for an example of fractionation of nucleic acids vs. proteins using ITP).

### Library preparation and quality control with SINC-seq

To critically evaluate SINC-seq, we performed experiments with 93 single cells of K562 human myeloid leukemia cells and generated 186 corresponding RNA-seq libraries using an off-chip Smart-seq2 protocol [26]. Ziegenhain et al. [27] recently reported a comprehensive comparison of scRNA-seq protocols including Drop-seq, Smart-seq with C1 (Fluidigm), and Smart-seq2. Among these methods, their work showed that Smart-seq2 is the most sensitive with the highest number of detected genes per cell. Further, Habib et al. [10, 28] recently reported a DroNc-seq platform approach which performs single-nucleus RNA-seq. The work demonstrated that DroNc-seq detected an average of 3295 and 5134 genes, respectively, for nuclei and cells of 3T3 cells. Here we have leveraged the sensitivity of the Smart-seq2 protocol and a full-length coverage to explore the retention of introns.

Both cytRNA-seq and nucRNA-seq of SINC-seq yielded 4.64 million reads per sample (Additional file 1: Figure S2b, c). The average transcriptomic alignments were 94 ± 1% (mean ± standard deviation (SD)) and 93 ± 1%, respectively, with cytRNA-seq and nucRNA-seq (Additional file 1: Figure S2d). Of the 93 single cells analyzed, all showed successful extraction as determined by monitoring the ionic current of the ITP process during extraction (Additional file 1: Figure S1c). Of these 93 single cells, 84 passed quality control (QC) for both cytRNA-seq and nucRNA-seq. Nine of the 93 cells failed the QC for either cytRNA-seq or nucRNA-seq. Further, in seven of the samples that failed QC, we observed low yield in the amplification of either cytRNA or nucRNA. In two of the samples, we observed incomplete fractionation. Thus, after the QC, we achieved 168 data sets consisting of 84 pairs of cytRNA-seq and nucRNA-seq (see Additional file 1: Supplementary Information section titled “Fractionation stringency”, Additional file 1: Figure S2, Additional file 3: Table S1, and Additional files 4 and 5).

We note that our protocol yielded smaller amounts of complementary DNA (cDNA) for extracted nucRNA than for cytRNA. The yield of cDNA with nucRNA was on par with that of single nuclei prepared with an off-the-shelf kit (PARIS Kit, Thermo Fisher Scientific) in which the cell membrane was lysed with a chemical agent. We thus hypothesize that the smaller amount of cDNA from the nucRNA fractions is due to the smaller amount of RNA in a nucleus compared to the cytRNA amount for the same cell. The total amount of cDNA per single cell was 26 ± 16% less than that obtained with a conventional single-cell protocol on average (Additional file 1: Figure S2a). We attribute this as mainly due to the loss at collecting cytRNA from the outlet well after ITP using a standard micropipette [17].

### SINC-seq dissects the difference in subcellular gene expression

To benchmark the technical aspects of SINC-seq, we assessed the sensitivity and repeatability of gene expression analyses with an in silico single-cell analysis. In this assessment, we used 56 pairs of nucRNA-seq and cytRNA-seq data taken with unperturbed K562 cells which were cultured under standard conditions (without NaB treatment). (See a comprehensive benchmark of SINC-seq in Additional file 1: Figures S3–S6 and the Supplementary Information section.) SINC-seq consistently detected 6210 ± 1400 (mean ± SD) and 5690 ± 1500 genes per cytRNA and nucRNA, respectively, and 8200 ± 1100 genes per cell with transcripts per million (TPM) greater than 1 (see Fig. 1d and comprehensive data in Additional file 1: Figure S3). SINC-seq also revealed that ~ 16% of transcripts were in the nucleus and ~ 84% in the cytoplasm (see Fig. 1e and also section titled “Computing cyt-normalized vs. nuc-normalized data: in silico single-cell normalization” in Methods) and also showed enriched expression of 226 and 3035 genes, respectively (Fig. 1f). Grindberg et al. [7] performed enrichment analyses with individually normalized reads per kilobase per million mapped reads (RPKM) values from nuclei and single cells. Compared to Grindberg’s work, our in silico single-cell approach uniquely enables quantitative comparison of gene expression and enrichment analyses between cytoplasm and nucleus. On average, SINC-seq displayed about a 5.3% smaller fraction of detected genes, 8070 ± 1100 genes at the sum total reads of 2.2 million (M) reads, than the conventional scRNA-seq, which detected 8520 ± 1100 genes (*n* = 12, 2.2 M reads). Again, we attribute this mainly to the losses associated with the collection of the cytRNA from the outlet well of the chip using a standard micropipette [17]. Notably, our in silico single-cell data showed a wider dynamic range in the detection of genes as compared to scRNA-seq (Additional file 1: Figure S4a). We attribute this improvement in the dynamic range of in silico single cells to the sequencing depth in nucRNA-seq. SINC-seq analyzed RNA expressions in the nucleus and cytoplasm individually with a similar number of sequencing reads. This means that nucRNA-seq has more depth per unit RNA template than cytRNA because of the smaller amount of nuclear RNA. This feature facilitates the detection of low abundant nuclear RNAs, hence resulting in a larger dynamic range for SINC-seq in silico cell analysis. To assess the validity of the gene detection with TPM < 1 in nucRNA-seq, we evaluated the detection of genes compared with bulk nucRNA-seq. Of 4235 genes with 0.01 < TPM < 1 in nucRNA-seq, bulk nucRNA-seq detected 4034 genes. This supports the conclusion that the detection of genes was not an artifact. We further evaluated this issue by analyzing with the coverages of the three lowest abundant genes (TPM~ 0.01) (Additional file 1: Figure S4b–d). Compared to conventional scRNA-seq, the in silico single-cell data showed a correlation with a mean coefficient of correlation *r* = 0.866 computed with a log-transformed expression (log10(TPM + 1)) (Fig. 1g). The nucRNA-seq also showed a correlation with scRNA-seq (different cells) with a mean coefficient of correlation *r* = 0.711, consistent with the results of Grindberg et al. [7]. Importantly, nucRNA-seq showed a higher correlation with its in silico single cell (same cells) than those with single cells (different cells), supporting the conclusion that the nucRNA-seq expression also reflects the transient gene expression of its respective single cell. This finding indicates that SINC-seq for the first time reveals the correlation of transient gene expression between nucRNA and cytRNA. Combined, an average gene expression profile of 12 in silico single cells (see Additional file 1: Figure S7 for the definition of 12 in silico single cells) showed an excellent matching with that of 12 scRNA-seq (Pearson correlation coefficient of *r* = 0.972, see Additional file 1: Figure S3q). The total number of detected genes (TPM > 1) in the 12 in silico single cells at an average sequencing depth of 2.2 M reads was 11,131, of which 9757 genes were also detected from 12 scRNA-seq averages (Additional file 1: Figure S3t). We again stress that the in silico normalization and resulting scRNA-seq is a novel method which uniquely leverages a physical separation and recovery as well as minimal cross-contamination enabled by our electrophoretic fractionation.

### Cell-cycle-related genes show correlated expression in cytoplasm and nucleus

**Fig. 2:**
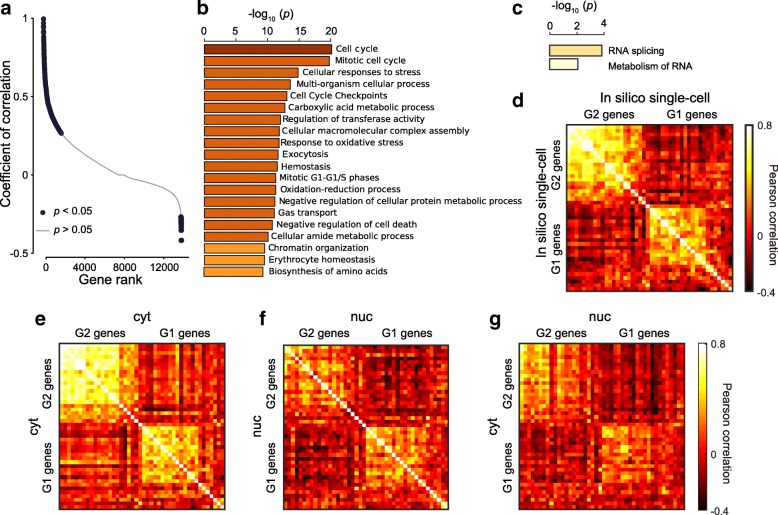
Landscape of cross-correlation between cytRNA and nucRNA unveiled transcriptional oscillation of cell-cycle genes in nucRNA highly correlated with expression in cytRNA. **a** Quantile plot of genes sorted in order of the coefficients of cross-correlation. **b** Gene ontology analysis with positively correlated genes (*p* < 0.05) in the quantile plot and **c** negatively correlated genes. **d**–**f** Cell-cycle genes in in silico single-cell data, cytRNA, and nucRNA show correlation with in-phase genes (G1 vs. G1 or G2 vs. G2) and negative correlation with out-of-phase genes (G1 vs. G2). List of genes of G1 and G2 phases are provided in Additional file 1: Figure S8. **g** Transcriptional oscillation of cell-cycle genes in nucRNA cross-correlated with the gene expression in cytRNA

To view the landscape of the correlation between nucRNA and cytRNA, we computed the cross-correlation of individual genes as a measure of covariation in the two subcellular compartments with 56 pairs of data taken with unperturbed K562 cells, and we ranked the genes by order of the value of the coefficient of correlation (Fig. 2a). We identified 1720 positively correlated genes and 29 negatively correlated genes with *p* < 0.05. Gene ontology analysis revealed that the correlated genes had cell cycle as an enriched function (Fig. 2b) and the negative correlation had RNA splicing (Fig. 2c). We note that identification of uncorrelated genes with this approach was technically challenging, as the method resulted in many uncorrelated genes with negligible significance (making it impossible to neglect the null hypothesis). In this section, we thus focused analyses on positively correlated and negatively correlated genes identifiable with statistical significance.

To dissect the correlated gene expression, we focused on cell cycle based on transcriptional oscillations [2] and phase-score analysis [3] (Additional file 1: Supplementary Information). Apart from the analysis of the correlation landscape, we obtained a list of cell-cycle genes [29] and extended an approach proposed by Klein et al. [2] to observe the behavior of individual cell-cycle genes. The in silico single-cell data showed the progression of the cell cycle with a correlated variation of in-phase genes and negative correlation of out-of-phase genes (G1 vs. G2) (Fig. 2d), consistent with scRNA-seq of K562 [2], and also with the progression of the phase score (Additional file 1: Figure S8a). Similarly, both cytRNA-seq and nucRNA-seq data revealed the cell cycle (Fig. 2e, f and Additional file 1: Figure S8b, c). Notably, we found that the negative correlation among out-of-phase genes was slightly higher in the nucRNA (*U* test, *p* = 8 × 10^− 26^, Additional file 1: Figure S8d). On the other hand, the correlation among in-phase genes was higher in the cytRNA, which may indicate further modulation in the cytoplasm.

To explore how the transcriptional oscillations in the nucRNA modulated the gene expression in the cytRNA, we extended the analyses to compute the cross-correlation between cytRNA and nucRNA with cell-cycle genes. The cell-cycle genes showed synchronized oscillation in each of the two subcellular compartments (Fig. 2g), consistent with the observation that the subpopulations segregated into the G1 and G2 groups showed corresponding up-regulation and down-regulation of G1 and G2 genes (Additional file 1: Figure S8e, f, and Supplementary Information). Together, these results suggest that the cytRNA and nucRNA have similar expression patterns of cell-cycle genes and that both of them solely have a potency to detect the cell cycle.

### Nuclear-retained introns attenuate the transcriptional oscillation

**Fig. 3:**
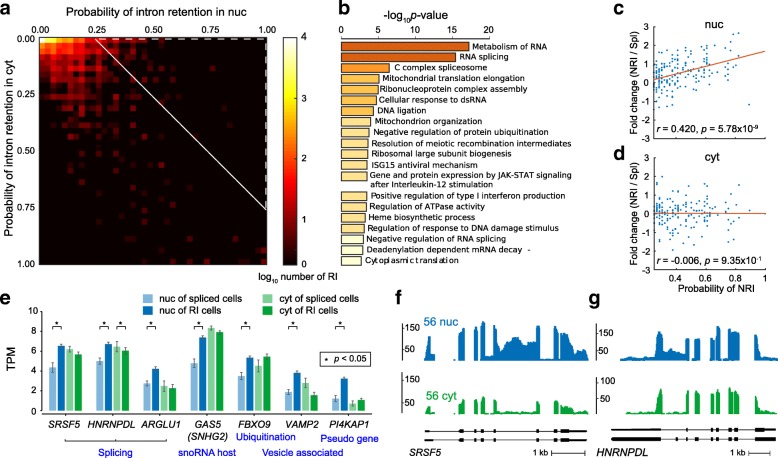
NRI-mediated attenuation of transcriptional oscillation in nucRNA. **a** Heatmap of the probability of RI in cytRNA and nucRNA fractions. NRI was identified in the *upper right* region indicated with the *broken white line*. **b** Gene ontology analysis with NRI, showing enriched functions like metabolism of RNA and RNA splicing. **c**, **d** Correlation analysis between the probability of NRI and the fold change of gene expression among cells with NRI and without NRI (*Spl* spliced) in nucRNA (*upper panel*) and cytRNA (*lower panel*), respectively. **e** Expression of seven genes that were highly regulated by NRI in nucRNA (*p* < 0.0003, Mann-Whitney *U* test), comparing with NRI vs. without NRI (Spl) in an individual fraction. **f** Coverage of *SRSF5* and **g** *HNRNPDL* genes showing higher intron reads in the nuclear fraction. Coverages of *ARGLU1*, *GAS5* (*SNHG2*), *FBXO9*, *VAMP2*, and *PI4KAP1* genes are provided in Additional file 1: Figure S10

We next studied the retained intron (RI)-mediated regulation of mRNA transport [30–32] leveraging the intron-rich reads with nucRNA-seq of SINC-seq (see Additional file 1: Figure S9 for comprehensive statistics on intron detection). After filtering RIs (Methods), SINC-seq detected 1760 ± 570 RIs per cell, of which 1290 ± 540 and 780 ± 290 were detected with nucRNA-seq and cytRNA-seq, respectively (Fig. 3a). We identified 242 nuclear-retained introns (NRIs) in 213 genes (Methods). Gene ontology analysis [33] determined that the 213 genes had enriched functions like metabolism of RNA and RNA splicing (Fig. 3b), consistent with previous studies [31, 34, 35]. We examined the relationship between the probability of NRI and gene expression in each individual (subcellular) fraction. We observed a positive correlation between them in the nucRNA (*r* = 0.42, *p* < 0.01, Fig. 3c), while there was no correlation in the cytRNA (*r* = − 0.006, *p* = 0.9, Fig. 3d). In contrast, we observed different enriched functions with cytoplasmic-enriched RIs (CRIs) and no correlation between probabilities of CRI and gene expression (Additional file 1: Figure S10a–c). For a better understanding of the function of NRIs, we examined the expression patterns of the top seven genes that were highly associated with NRI-mediated regulation (Fig. 3e–g and Additional file 1: Figure S10d–h). Notably, the seven genes contained three splicing-related genes [36] and a small nucleolar RNA (snoRNA) host gene [37]. These data lead us to hypothesize that the NRIs likely attenuate the transcriptional oscillation in the nucleus via fine-tuning of the RNA metabolism in order to maintain the gene expression and functional RNAs in the cytoplasm.

### Sodium butyrate treatment on K562 drives diverging gene expression in subcellular compartments

**Fig. 4:**
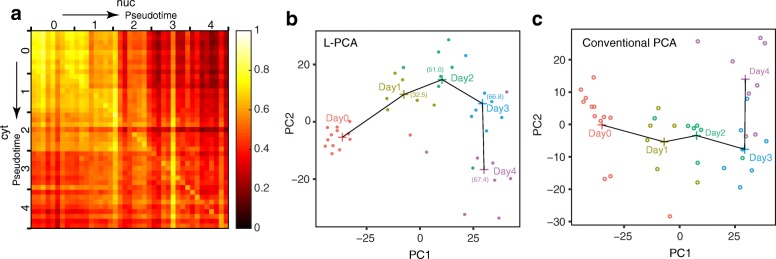
Differentiation of K562 cells to erythroid cells shows a dynamical change of cross-correlation between cytRNA and nucRNA. **a** The cross-correlation of DEG expression between cytRNA and nucRNA along with the pseudotime. Numbers along axes show groups of cells used in the analysis shown in Additional file 1: Figure S12b. **b** L-PCA analysis of differentiating K562 cells to erythroid cells by sodium butyrate treatment. **c** Conventional PCA analysis with differentiating K562 cells

To explore the correlation dynamics under perturbation, we further performed SINC-seq by differentiating K562 cells along the erythroid lineage with NaB (see Methods), sampling 8–13 cells per day over 5 days. We used NaB to modulate the histone acetylation process and observed transient effects of the epigenetic modification on the correlation through the changes in transcriptional activation and export of mRNA to the cytoplasm. With 41 successful SINC-seq data sets (82 RNA-seq data in total), we detected differentially expressed genes (DEGs); 264 up-regulated and 177 down-regulated genes were in cytRNA, and 64 up-regulated and 2 down-regulated genes were in nucRNA (Additional file 1: Figure S11). We examined the dynamics of the cross-correlation of DEG expression between cytRNA and nucRNA along the differentiation by ordering cells with the pseudotime computed using Monocle (version 2.4.0) [38, 39] to in silico single-cell data (Fig. 4a, Additional file 1: Figure S12a). To provide the statistical significance, we divided cells into five groups with the pseudotime (see the divisions in Fig. 4a) and evaluated the cross-correlation of gene expression between nucRNA and cytRNA (Additional file 1: Figure S12b). The cross-correlation between cytRNA and nucRNA exhibited a gradual decrease with increasing pseudotime, suggesting that the subcellular gene expression patterns behaved differently and diverged as the differentiation proceeded. We also found that non-DEGs showed a less significant change in the coefficient of cross-correlation with the differentiation (Additional file 1: Figure S12c). We further explored this observation with the control experiments (Additional file 1: Figure S12c–e). We found that the cross-correlation with DEGs showed no apparent change with unperturbed cells along the pseudotime (Additional file 1: Figure S12d). These findings and data with population controls at day 0 and day 4 (Additional file 1: Figure S12e) indicate that the change in the cross-correlation was not an artifact but reflects the dynamics of cytRNA and nucRNA along the differentiation. Notably, the cross-correlation between nucRNA on day 4 and cytRNA on day 1 (near the top right corner in Fig. 4a) was lower compared to that between nucRNA on day 1 and cytRNA on day 4 (near the bottom left corner), suggesting that nucRNA was the driver of the diverging gene expression.

We hypothesize that the divergence of gene expressions in the two subcellular compartments reflects the transient responses of different regulatory pathways in cytRNA and nucRNA. To leverage the different behaviors of nucRNA and cytRNA, we introduced a localization-embedded principal component analysis (L-PCA) that computed principal components (PCs) with the subcellular gene expressions of DEGs (Fig. 4b). In the L-PCA, we treated genes in cytRNA and nucRNA as different ones and represented a single cell as a vector having double dimension instead of using the conventional approach. As expected, L-PCA resolved the trajectory of the differentiation slightly differently compared to a conventional PCA that computed PCs with in silico single-cell data (Fig. 4c). To further corroborate the L-PCA, we performed PCA on data sets of an individual fraction, showing this specific and unique transient in the nucRNA — that is the day 3 cluster was furthest from the day 0 cluster in nucRNA (Additional file 1: Figure S12f, g). With our data set, it was difficult to conclude whether L-PCA can offer better clustering than conventional PCA. However, these results at least demonstrated that the L-PCA with SINC-seq is practical and potentially useful when analyzing a biological process involving regulations at multiple layers of single cells.

## Discussion

A fundamental question is how the transcriptional oscillation in the nucRNA, which is inherently stochastic, is transported to and correlated with gene expression in the cytRNA. SINC-seq enabled direct and quantitative comparison of gene expressions between a nucleus, a cytoplasm, and a whole cell of the same single cell. This comparison reveals that the cell may conceivably fine-tune a portion of its expression upon transport to the cytoplasm (e.g., with NRI genes), while preserving correlation of other portions of its expression upon transport (e.g., with cell-cycle-related genes). SINC-seq also revealed that the cells under external perturbation dynamically alter the correlation and exhibit a unique trajectory of differentiation at subcellular resolution. These findings shed new light on the characteristics of post-transcriptional regulations with a single cell and subcellular compartment resolution.

Our study also suggests a compelling caution to an approach that approximates the transcriptomic profile of the whole cell with that of a single compartment without validation. The SINC-seq platform will be broadly applicable to different types of cells as long as they are isolated as singles. The method thus will contribute to validate existing subcellular RNA-seq methods [4, 5, 9–11, 19, 20] and also define their limitations.

## Conclusion

To dissect transcriptional correlation in subcellular compartments, we devised SINC-seq, which enables integrated nuclear and cytoplasmic RNA-seq of single cells by coupling physical fractionation of cytRNA from the nucleus of a single cell with a high-throughput RNA-seq. Leveraging SINC-seq, we explored the landscape of the correlation between nucRNA and cytRNA with a total of 84 K562 cells, which corresponds to 168 RNA-seq libraries. The SINC-seq data unveiled three distinct natures of correlation among cytRNA and nucRNA that reflected the physiological state of single cells: highly correlated expression in cell-cycle-related genes, the distorted correlation via NRIs, and the correlation dynamics along the differentiation of K562 cells to erythroid cells under sodium butyrate perturbation. These data provide unique insights into the regulatory network of mRNA from the nucleus toward the cytoplasm at the single-cell level.

## Methods

### Cells

We purchased K562 cells (human lymphoblast, chronic myelogenous leukemia) from RIKEN BioResource Center and the Japanese Collection of Research Bioresources (JCRB) cell bank. We cultured the K562 cells in RPMI-1640 Medium (Life Technologies) with 10% fetal bovine serum and 1% penicillin-streptomycin-glutamine at 37 °C in 5% CO_2_. We washed the cells with phosphate-buffered saline once and suspended them in a sample buffer containing 50 mM imidazole, 25 mM 4-(2-hydroxyethyl)-1-piperazineethanesulfonic acid (HEPES), and 175 mM sucrose (pH 7.6) at a concentration of ~ 0.8 cells/μL and stored them on ice until the experiments were performed. To differentiate the K562 cells, we incubated them with 1 mM NaB (Sigma-Aldrich, B5887) and harvested them after 96 h of induction.

### Buffers

We designed buffers for ITP-based selective extraction, separation (from the trapped nucleus), purification, and transport of cytRNA to the cytRNA output well of the chip (see more details in Shintaku et al. [16] and Kuriyama et al. [17]). The leading electrolyte buffer (LE) components were 50 mM Tris and 25 mM HCl containing 0.4% poly(vinylpyrrolidone) (PVP) (calculated pH of 8.1). The trailing electrolyte buffer (TE) components were 50 mM imidazole and 25 mM HEPES containing (initial calculated pH of 8.3) 0.4% PVP. We included PVP to suppress electroosmotic flow. We purchased Tris, HEPES, imidazole, and HCl from Sigma-Aldrich, and PVP (molecular weight 1 MDa) from Polyscience. We prepared all solutions in UltraPure DNase-/RNase-free deionized (DI) water (Life Technologies).

### Microfluidic system setup

We fabricated polydimethylsiloxane (PDMS, Sylgard 184, Dow Corning) microchannel superstructures (Additional file 1: Figure S1a, b) with a soft lithography and bonded them to a glass substrate. SU-8 (SU-8 2025, MicroChem) molds were prepared on glass substrates with the microchannel patterns made of chromium thin films, exposing the SU-8 to ultraviolet light through the pattern. The nominal channel width and depth of the microchannels were 50 μm and 25 μm, respectively. We designed 3-μm-wide and 5-μm-long hydrodynamic traps. We have optimized the size of the hydrodynamic trap as 3 μm wide and 25 μm deep so that it can capture a single cell and trap a nucleus during the electric field extraction of cytRNA. We observed some deformation of cells due to the presence of the pressure-driven flow, but observed no mechanical lyses prior to the application of the electric field. This trapping process had a timescale of several minutes.

Before each experiment, we preconditioned the microchannel by filling the inlet and outlet wells with washing solutions and applying a vacuum to the waste well. Our washing process was as follows: 1 M NaOH for 1 min, 1 M HCl for 1 min, and DI water for 1 min. All washing solutions contained 0.1% Triton X-100 to suppress bubble clogging in the hydrophobic microchannel.

Following this, we loaded 9.5 μL of LE and TE to the outlet and inlet wells, respectively, and briefly applied a vacuum to the waste well to exchange the solution in the microchannel with LE and TE. The hydrodynamic pressure induced by buffers in the inlet and outlet wells created a pressure-driven laminar flow from both inlet and outlet wells toward the waste well and formed a stable LE-TE interface at the junction of three microchannels. We then picked up a single cell of interest from the cell suspension by aspirating 1 μL using a standard micropipette. We observed this process using a microscope and confirmed aspiration of a single cell. We then dispensed this 1-μL volume into the inlet well and introduced it into the microchannel via the pressure-driven flow. Once we visually confirmed the captured single cell at the hydrodynamic trap (Fig. 1c), we added 9.5 μL of the TE to the waste well to reduce the pressure-driven flow. We aborted our protocol in cases where we observed two or more cells at the hydrodynamic trap. We placed 300-μm-diameter platinum wire electrodes into the wells and applied − 150 V, − 170 V, and 0 V to the electrodes at the inlet, waste, and outlet wells, respectively. The DC voltage created a concentrated electric field at the hydrodynamic trap (Additional file 1: Figure S1d) and lysed the cytoplasmic membrane within 1 s. Appropriate placement of the ITP buffers with the DC electric field enabled an immediate transition from the lysis to an ITP process that collects and focuses cytRNA into an ITP-zone, TE-to-LE interface. At 40 s, we changed the voltages to − 350 V and − 510 V at the inlet and waste wells, respectively, to accelerate the migration of the ITP zone. The ITP zone transported the cytRNA to the output well in about 100 s, while the nucleus was retained at the hydrodynamic trap. We also monitored the current during the extraction with a computer running a custom MATLAB (Mathworks, Inc., Natick, MA, USA) script. The magnitude of the current decreased as the ITP zone (containing the focused cytRNA) advanced in the channel and as the lower conductivity TE replaced the higher conductivity LE (Additional file 1: Figure S1c). The current signal plateaued near *t* = 100 s, coincident with the time at which the focused cytRNA eluted into the outlet well. We deactivated the voltages at 200 s and used a standard pipette to transfer two aliquots from the chip: 9.5 μL from the outlet well containing the cytRNA and 1 μL containing the cell nucleus from the inlet well. Detailed descriptions of a similar protocol and chip, together with a narrated video description, were reported by Kuriyama et al. [18].

### Library preparation and mapping analysis

We synthesized respective cDNA libraries from the fractionated cytRNA and nucRNA separately using Smart-seq2 (SMART-seq v4 Ultra Low Input RNA Kit for Sequencing, Clontech) with 18 polymerase chain reaction (PCR) cycles followed by purification with Agencourt AMPure XP (Beckman Coulter). We examined the yield and quality of cDNA, respectively, with a Qubit 2.0 Fluorometer (Thermo Fisher Scientific) and quantitative PCR (qPCR) targeting *GAPDH* (glyceraldehyde-3-phosphate dehydrogenase, Hs02758991_g1, Thermo Fisher Scientific) and *HBG* (gamma-globin genes, Hs00361131_g1, Thermo Fisher Scientific). We performed the tagmentation reaction with 200 pg cDNA using a Nextera XT DNA Sample Preparation Kit (Illumina) and purified the cDNA library following the manufacturer’s protocol, except that we eluted the cDNA sample with 24 μL instead of 50 μL (see Additional file 1: Figure S2a for yields of cDNA). We pooled 98–108 libraries and sequenced them on an Illumina HiSeq 2500 with 100-base paired-end reads to an average depth of 4.64 million reads (Additional file 1: Figure S2b, c). We mapped the trimmed sequencing reads to the transcripts derived from the human reference genome (GRCh37.75) using the STAR (version 2.5.1b) mapping program [40] with ENCODE options, and calculated expression estimates with TPM using RNA-Seq by Expectation-Maximization (RSEM v1.3.0) [41].

We performed DE analyses individually with cytRNA and nucRNA comparing day 1–day 4 samples to day 0 samples. We used the MATLAB functions “nbintest” and “mafdr” and identified significance with *p* values less than 0.001 and absolute log2 fold changes greater than unity.

### Analysis of intron retention

We computed intron expressions with fragments per kilobase of intron per million mapped reads (FPKM) using 347,041 unique introns (longer than 50 nt) on the genome annotation with 56 SINC-seq data of K562 cells under standard culturing conditions. On average, SINC-seq yielded 14.8% reads mapped to introns with nucRNA-seq, but only 1.1% with cytRNA-seq. Further, SINC-seq detected 38,800 ± 10,000 and 34,800 ± 8000 unique introns in nucRNA-seq and cytRNA-seq, respectively, and 56,300 ± 9500 per cell with FPKM of more than 0. We identified an RI that had at least 10% expression level of the gene, 95% coverage in the intronic region, and non-zero expression in the adjacent exon. We discarded intron reads locating on a gene with less than 2 TPM. On the other hand, we identified a fully spliced intron that had less than 1% expression of the gene and 50% coverage in the adjacent exon. We discarded intron reads that failed the preceding criteria. We validated the RI identification with splice site scores [42], which showed lower values with RIs than fully spliced introns, using 9mer (exonic 3mer + intronic 6mer) around the 5′ splice site (*p* value < 2.2 × 10^− 16^, *U* test), and 23mer (intronic 20mer + exonic 3mer) around the 3′ splice site (*p* value < 2.5 × 10^− 12^, *U* test). In total, we detected 17,277 RIs, of which 14,134 and 2316 RIs had a higher probability of RI in nucRNA and cytRNA, respectively. The RI enrichment in the nucleus was consistent with that for previous studies [30–32].

To identify NRIs, we calculated the probability of intron retention defined as the proportion of cells with the RI and identified NRIs that had 0.25 higher probability in the nuclear fraction than in the cytoplasmic fraction. We then filtered unique NRIs, discarding smaller NRIs that had overlap with a long NRI. On the other hand, we identified CRIs that had 0.25 higher probability in the cytoplasmic fraction than in the nuclear fraction.

### Computing cyt-normalized vs. nuc-normalized data: in silico single-cell normalization

We computed in silico single-cell RNA-seq (scRNA-seq) data with cytRNA-seq and nucRNA-seq data, scaling the raw TPM values and combining the cytRNA-seq and nucRNA-seq data as

S1 $$ {TPM}_{in- silico}={TPM}_{cyt}+{TPM}_{nuc}, $$

S2 $$ {TPM}_{cyt}=\alpha {TPM}^{\ast }, $$

S3 $$ {TPM}_{nuc}=\beta {TPM}^{\ast }, $$

where *α* and *β* are normalization factors, which make the summation of the TPM values, *TPM*_*in silico*_, of the in silico single-cell data to be one million. We here write raw TPM values with an asterisk and TPM values of in silico single-cell data, cytRNA-seq, and nucRNA-seq, respectively, with their subscripts. We calculate *α* and *β* using Δ*Ct* of qPCR data taken at the QC of cDNA (Additional file 3: Table S1) as

S4 $$ \alpha =\frac{2^{\Delta Ct}}{\frac{{TPM^{\ast}}_{cyt, geneA}}{{TPM^{\ast}}_{nuc, geneA}}+{2}^{\Delta Ct}}, $$

S5 $$ \beta =1-\alpha, $$

where TPM^*^_cyt,geneA_, TPM^*^_nuc, geneA_, and Δ*Ct* are, respectively, the raw TPM value of *gene A* with cytRNA-seq, the raw TPM value of *gene A* with nucRNA-seq, and Δ*Ct* = *Ct*_nuc, geneA_ – *Ct*_cyt, geneA,_ which is Δ*Ct* with respect to *gene A*. We calculated pairs of *α* and *β* with *GAPDH* and *HBG* genes (*HBG1* + *HBG2*) as *gene A*s, and used the mean *α* and *β* to compute the *TPM*.

We here note that *α* and *β*, respectively, indicate the (complementary) fractions of cytoplasmic transcripts and nuclear transcripts in the in silico single cells. We thus can quantify the fraction of the cytoplasmic transcript as shown in Fig. 1e.

### L-PCA

The PCA for the conventional scRNA-seq uses an (*n* × *m*) matrix of gene expressions (*n* = number of genes) with multiple samples (*m* = number of samples); however, our L-PCA uses a (2*n* × *m*) matrix of gene expressions, having a twofold dimension compared to the conventional matrix, derived from cytRNA-seq and nucRNA-seq. The L-PCA was performed using PCA with “prcomp” in R.

## Additional files

Additional file 1: Supplementary information and **Figures S1–S12.** (DOCX 4587 kb)

Additional file 3: **Table S1.** Quality control of SINC-seq samples. (XLSX 21 kb)

Additional file 4: **Movie S2.** Experimental run #55 with unsuccessful fractionation. (MP4 1372 kb)

Additional file 5: **Movie S3.** Experimental run #69 with unsuccessful fractionation. (MP4 1116 kb)

## References

[1] Wu AR (2014). Quantitative assessment of single-cell RNA-sequencing methods. Nat Methods.

[2] Klein AM (2015). Droplet barcoding for single-cell transcriptomics applied to embryonic stem cells. Cell.

[3] Macosko EZ (2015). Highly parallel genome-wide expression profiling of individual cells using nanoliter droplets. Cell.

[4] Habib N (2016). Div-Seq: single-nucleus RNA-Seq reveals dynamics of rare adult newborn neurons. Science.

[5] Lake BB (2016). Neuronal subtypes and diversity revealed by single-nucleus RNA sequencing of the human brain. Science.

[6] Tanay A, Regev A (2017). Scaling single-cell genomics from phenomenology to mechanism. Nature.

[7] Grindberg RV (2013). RNA-sequencing from single nuclei. Proc Natl Acad Sci U S A.

[8] Krishnaswami SR (2016). Using single nuclei for RNA-seq to capture the transcriptome of postmortem neurons. Nat Protoc.

[9] Lacar B (2016). Nuclear RNA-seq of single neurons reveals molecular signatures of activation. Nat Commun.

[10] Habib N (2017). Massively parallel single-nucleus RNA-seq with DroNc-seq. Nat Methods.

[11] Gao R (2017). Nanogrid single-nucleus RNA sequencing reveals phenotypic diversity in breast cancer. Nat Commun.

[12] Macaulay IC (2015). G&T-seq: parallel sequencing of single-cell genomes and transcriptomes. Nat Methods.

[13] Angermueller C (2016). Parallel single-cell sequencing links transcriptional and epigenetic heterogeneity. Nat Methods.

[14] Cheow LF (2016). Single-cell multimodal profiling reveals cellular epigenetic heterogeneity. Nat Methods.

[15] Han L (2014). Co-detection and sequencing of genes and transcripts from the same single cells facilitated by a microfluidics platform. Sci Rep.

[16] Shintaku H (2014). On-chip separation and analysis of RNA and DNA from single cells. Anal Chem.

[17] Kuriyama K, Shintaku H, Santiago JG (2015). Isotachophoresis for fractionation and recovery of cytoplasmic RNA and nucleus from single cells. Electrophoresis.

[18] Kuriyama K, Shintaku H, Santiago JG (2016). Protocol for microfluidic system to automate the preparation and fractionation of the nucleic acids in the cytoplasm versus nuclei of single cells. Bio-protocol.

[19] Hou Y (2016). Single-cell triple omics sequencing reveals genetic, epigenetic, and transcriptomic heterogeneity in hepatocellular carcinomas. Cell Res.

[20] Hu Y (2016). Simultaneous profiling of transcriptome and DNA methylome from a single cell. Genome Biol.

[21] Kohler A, Hurt E (2007). Exporting RNA from the nucleus to the cytoplasm. Nat Rev Mol Cell Biol.

[22] Gerstberger S, Hafner M, Tuschl T (2014). A census of human RNA-binding proteins. Nat Rev Genet.

[23] Wang J, Lu C (2010). Kinetics of NF-κB nucleocytoplasmic transport probed by single-cell screening without imaging. Lab Chip.

[24] Zhan Y, Lu C (2010). One-step extraction of subcellular proteins from eukaryotic cells. Lab Chip.

[25] Qu, Marshall LA, Santiago JG (2014). Simultaneous purification and fractionation of nucleic acids and proteins from complex samples using bidirectional isotachophoresis. Anal Chem.

[26] Picelli S (2013). Smart-seq2 for sensitive full-length transcriptome profiling in single cells. Nat Methods.

[27] Ziegenhain C (2017). Comparative analysis of single-cell RNA sequencing methods. Mol Cell.

[28] Habib N, et al. DroNc-Seq: deciphering cell types in human archived brain tissues by massively-parallel single nucleus RNA-seq. bioRxiv. 2017; 10.1101/115196

[29] Whitfield ML (2002). Identification of genes periodically expressed in the human cell cycle and their expression in tumors. Mol Biol Cell.

[30] Ninomiya K, Kataoka N, Hagiwara M (2011). Stress-responsive maturation of Clk1/4 pre-mRNAs promotes phosphorylation of SR splicing factor. J Cell Biol.

[31] Boutz PL, Bhutkar A, Sharp PA (2015). Detained introns are a novel, widespread class of post-transcriptionally spliced introns. Genes Dev.

[32] Naro C (2017). An orchestrated intron retention program in meiosis controls timely usage of transcripts during germ cell differentiation. Dev Cell.

[33] Tripathi S (2015). Meta- and orthogonal integration of influenza “OMICs” data defines a role for UBR4 in virus budding. Cell Host Microbe.

[34] Iida K, Go M (2006). Survey of conserved alternative splicing events of mRNAs encoding SR proteins in land plants. Mol Biol Evol.

[35] Hsu TY (2015). The spliceosome is a therapeutic vulnerability in MYC-driven cancer. Nature.

[36] Lareau LF (2007). Unproductive splicing of SR genes associated with highly conserved and ultraconserved DNA elements. Nature.

[37] Williams GT, Farzaneh F (2012). Are snoRNAs and snoRNA host genes new players in cancer?. Nat Rev Cancer.

[38] Qiu X (2017). Single-cell mRNA quantification and differential analysis with Census. Nat Methods.

[39] Trapnell C (2014). The dynamics and regulators of cell fate decisions are revealed by pseudotemporal ordering of single cells. Nat Biotechnol.

[40] Dobin A (2013). STAR: ultrafast universal RNA-seq aligner. Bioinformatics.

[41] Li B, Dewey CN (2011). RSEM: accurate transcript quantification from RNA-Seq data with or without a reference genome. BMC Bioinformatics.

[42] Yeo G, Burge CB (2004). Maximum entropy modeling of short sequence motifs with applications to RNA splicing signals. J Comput Biol.

[43] Abdelmoez MN (2017). Gene expressions in nucleus and cytoplasm at the single cell resolution. Sequencing Read Archive.

