# Supplementary material for: SINC-seq: correlation of transient gene expressions between nucleus and cytoplasm reflects single-cell physiology
Source: Genome Biol. 2018 Jun 6;19:66. doi: 10.1186/s13059-018-1446-9 (PMC5989370; doi:10.1186/s13059-018-1446-9)
Supplement: Supplementary file 2 — Supplementary information and Figures S1–S12. (DOCX 4587 kb) [file 13059_2018_1446_MOESM1_ESM.docx]

Supplementary Information and Figures

SINC-seq: correlation of transient gene expressions between nucleus and cytoplasm reflects single-cell physiology

Mahmoud N. Abdelmoez,^1,2¶^ Kei Iida,^3¶^ Yusuke Oguchi,^4¶^ Hidekazu Nishikii,^5^ Ryuji Yokokawa,^1^ Hidetoshi Kotera,^1^ Sotaro Uemura,^4^ Juan G. Santiago,^6^ and Hirofumi Shintaku^*1,2^

^1^Department of Micro Engineering, Graduate School of Engineering, Kyoto University; ^2^Microfluidics RIKEN Hakubi Research Team, RIKEN Cluster for Pioneering Research; ^3^Medical Research Support Center, Graduate School of Medicine, Kyoto University; ^4^Department of Biological Sciences, Graduate School of Science, The University of Tokyo; ^5^Department of Hematology, Faculty of Medicine, University of Tsukuba; ^6^Department of Mechanical Engineering, Stanford University

*Corresponding author: hirofumi.shintaku@riken.jp

^¶^These authors contributed equally to this work.

## Fractionation stringency

To assess the fractionation stringency of cytRNA versus nucRNA, we investigated the expression of mitochondrial RNA and nuclear-enriched RNA (*MALAT1*, *SON*, and *XIST*) [1-3] in individual fractions (Fig. S2e, f). SINC-seq showed that the protocol extracts an average of 99% of mitochondrial RNA to the outlet well as cytRNA(Figs. S2g, h). Of 86 SINC-seq data, two (denoted as 55 and 69 in Figs. S2e, f) displayed incomplete fractionation with respectively lower and higher expressions of mitochondrial RNAs in cytRNA and nucRNA. In these two cases, we observed that the nuclei left the hydrodynamic trap and moved toward the inlet well upon the application of the electric field (Additional files 4 and 5). This evidence indicates that the mitochondrial RNA extraction for these two cells was incomplete given the short residence time of the cell in the trap.

We further note that nucRNA-seq of the SINC-seq detected an average of 91.3% of the nuclear-enriched RNA for all the cells analyzed (Fig. S2g, h). Interestingly, *XIST* showed the highest localization in nucRNA with an average of 97.5±9.3% expression among the three nuclear-enriched RNAs, whereas *MALAT1* and *SON*, which are non-coding and protein-coding genes, respectively, showed 83.8±16% and 77.1±23% expression in nucRNA. We hypothesize that these different degrees of localization reflect their biological functions as the degree of localization showed uncorrelated behavior while the degrees of cytoplasmic localization of mitochondrial RNAs showed a significant correlation (*r*=0.93). Further, as the translocation of *MALAT1* to cytoplasm has been reported [3], we think *XIST* would be the best biomarker to quantify the leaked nuclear RNA during the extraction.

## Reproducibility and sensitivity of gene detection with SINC-seq

To benchmark the sensitivity and repeatability of gene detection, we analyzed cytRNA-seq and nucRNA-seq of SINC-seq with a similar approach proposed by Wu et al. [4]. Briefly, we assessed the reproducibility of each RNA-seq by the number of genes detected in all combinations of replicate sample pairs. We determined sensitivity by calculating how many genes were detected by each RNA-seq compared to matching population controls created with bulk cytRNA and nucRNA, respectively. In this assessment, we used 56 pairs of nucRNA-seq and cytRNA-seq data taken with K562 cells under a standard culturing condition (without NaB treatment) and population controls prepared with 200 pg of bulk cytRNA and bulk nucRNA extracted from the cell population (see the section “Bulk cytRNA-seq and bulk nucRNA-seq with cell population” in Supplementary Information).

To assess the reproducibility of cytRNA-seq and nucRNA-seq of SINC-seq, we computed the number of overlapping genes in all combinations of replicate sample pairs (Fig. S3f, g). The cytRNA-seq (Fig. S3f) reproductively detected 4,400±1,100 genes, which were computed as 71% of the mean number of detected. The nucRNA-seq (Fig. S3g) showed less reproducibility with 3,420±1,000 of detected genes, which were computed as 60% of the mean number of genes detected. The cytRNA-seq also showed higher coefficients of pair-wise correlation of gene expression as 0.824±0.058 than the nucRNA-seq as 0.631±0.080 (Fig. S3h, i).

To further benchmark our nucRNA-seq, we compared our data to single nuclei RNA-seq, for which we isolated nuclei with PARIS Kit (Thermo Fisher Scientific) and prepared the single nuclei RNA-seq libraries with the same Smart-seq2 protocol (see the Supplementary section of Single nuclei RNA-seq with the off-the-shelf kit). The comparison showed a non-significant difference in the number of detected genes and reproducible results, while the coefficient of the correlation was lower with nucRNA-seq of SINC-seq than with single nuclei RNA-seq (Fig. S5d). The integrated data of 12 nucRNA-seq detected 12,400±23(S.E.) genes and that of 12 single nuclei RNA-seq did 12,100 genes, of which 10,300±10(S.E.) genes were detected by both methods (Fig. S5e). The analyses of the differentially expressed genes showed that SINC-seq yielded 76 of over-expressed genes and 40 of under-expressed genes compared to single nuclei RNA-seq with a threshold of p<0.001. Of 40 under-expressed genes, three mitochondrial RNAs showed under-expression with about 6.2 folds in nucRNA-seq of SINC-seq, indicating less contamination from cytoplasmic RNA. The nuclear-enriched RNAs (*MALAT1*, *SON*, and *XIST*) and the most of snoRNAs showed the insignificant difference in the expression level, suggesting no significant leakage of nuclear RNA with SINC-seq method (Fig. S5.f). Hence, taken together, nucRNA-seq of SINC-seq offer a sensitivity similar to single nuclei RNA-seq and with less contamination from cytoplasmic RNA.

To uncover the characteristics of cytRNA and nucRNA, we created pseudo bulks, 56 cytRNA-seq and 56 nucRNA-seq, randomly sampling reads respectively from 56 of cytRNA-seq and 56 of nucRNA-seq data to be ~4 million reads, and compared the gene expression patterns (Fig. S3r, s) and the number of detected genes with respective matching population control (Fig. S3 u, v). Strikingly, the 56 nucRNA-seq yielded a larger number of detected genes and a lower coefficient of correlation than the 56 cytRNA-seq to the corresponding population controls. These observations reproduced characteristics of the population controls (bulk cytRNA-seq: 11,550 detected genes (TPM>1), *r* =0.969±0.006(*n*=6); bulk nucRNA-seq: 13,398 detected genes, *r* = 0.883±0.022(*n*=6)), indicating that the nucRNA had richer transcriptomic heterogeneity than cytRNA.

## Single nuclei RNA-seq with off-the-shelf kit

To obtain control data, we performed single nuclei RNA-seq. In these experiments, we isolated nuclei chemically lysing cytoplasmic membranes and washed the nuclei as per the product’s protocol (PARIS Kit, Thermo Fisher Scientific). We prepared single nuclear RNA-seq libraries with the Smart-seq2 protocol in a tube and performed the sequencing with the MiSeq platform. In this sequencing run, we pooled 12 control samples and 13 nucRNA-seq samples of SINC-seq for the comparison and obtained 75 nt long paired-end reads to be an average of 1.7 M reads per sample. We confirmed that the RNA-seq with MiSeq platform could provide a good approximation of two characteristics of the SINC-seq data obtained with HiSeq2500 platform. First, the gene expression pattern compares well (*r*=0.98±5e-4 (S.E., *n*=13)) and, second, the number of detected genes compares well (see Figs. S5a, b).

## Validation of scaling

We assessed the scaling strategy comparing ERCC spike-in expressions between cytRNA-seq and nucRNA-seq (Fig. S6a-c). We found that the scaled ERCC expressions that computed with normalization factors (Fig. S6b) displayed significantly less biased result than the raw ERCC expressions (Fig. S6a). As conventional normalization with ERCC, the estimated counts (Fig. S6c) were also converted from raw TPM values and displayed the less biased result.

We also analyzed the relationship between variance versus mean expressions (Fig. S6d-f) to evaluate the relationship of CV^2^- μ (CV and μ are the coefficient of variation and the mean number of molecules per reaction, respectively). We found that the TPM (Fig. S6e) and the estimated counts (Fig. S6f) displayed similar noise level among cytRNA-seq and nucRNA-seq especially at the Poissonian regime, which was less than 10^0^ molecule per reaction. We also found that the baseline of the CV^2^ of ERCC (black points) roughly agreed with the Poissonian noise (a broken line in Fig. S6f).

To investigate the quantification bias in in silico single cell normalization, we compared the expression levels of genes enriched in either cytRNA or nucRNA to those with scRNA-seq. We selected top 20 genes enriched in cytRNA and nucRNA, respectively, which are most susceptible to be biased (Fig. S6). Overall, the gene expression pattern displayed a high correlation (*r*=0.872) and a low bias with a mean slope of 0.90 (Fig. S6i, j). These findings validated our scaling strategy and quantitative comparison between cytRNA and nucRNA-seq with the TPM.

## Bulk cyt RNA-seq and bulk nuc RNA-seq with cell population

To obtain population control of RNA-seq, we performed cytoplasmic and nuclear RNA extraction with an off-the-shelf kit (PARIS kit, Thermo Fisher Scientific) following the manufacturer's protocol. We prepared cDNA samples of cytoplasmic and nuclear RNA with 200 pg RNA using the same protocol with the single cell samples, except for using 15 PCR cycles. We performed tagmentation and fragmentation with 150 pg cDNA using Nextera XT DNA sample kit and following the manufacturer's protocol.

To quantify the RNA masses in individual fractions, we measured the RNA masses with NanoDrop (NanoDrop Lite UV-Vis Spectrophotometer, ThermoFisher Scientific). To remove the genomic DNA in nuclear RNA, we performed the lithium chloride precipitation, which removed 95.0±1.3%(mean±95% confidence interval, n=4) of DNA.

## Transcriptional oscillation of cell-cycle-related genes

To test whether the SINC-seq might capture transcriptional oscillation due to cell-cycle, we examined Pearson correlation with the variation of 44 genes previously categorized to a particular cell-cycle phase [5]. Genes of the individual group (G1 or G2 phase) were ordered with hierarchical clustering (Gene names are listed in Fig. S8f). Here we note that for cytRNA and nucRNA raw TPM values were used (Fig. 2b-d) and the gene orders correspond with that determined in the in silico single-cell data.

## Cell-cycle analysis with phase-score

To evaluate cell-cycle, we calculated the phase-score (G1/S, S, G2/M, M, M/G1) of each sample as follows. First, as described in Macosko et al. [6], we extracted gene sets showing a correlation with *r* > 0.3 between the expression pattern of each gene and the average expression pattern of all genes in the respective phase. Then, we averaged the expression levels (log2(TPM+1)), followed by the two-step normalization. First, the scores were normalized for each phase, then normalized for each cell, so that we obtained the normalized phase score for each sample and assigned a cell-cycle phase with the maximum phase score. Normalized phase scores were shown as a heatmap (Figs. S8a-c) and were ordered by descending order of phase score in respective phase. Here we also note that this analysis was performed by independently in silico single-cell (Fig. S8a), cytRNA (Fig. S8b) and nucRNA (Fig. S8c). We note that for cytRNA and nucRNA raw TPM values were used.

## Clustering and characterizing cells in G1 and G2 groups

To distinguish cells with cell-cycle phases, we performed a clustering analysis of the in silico single cell data applying t-distributed stochastic neighbor embedding (tSNE) to the phase-scores. Cells segregated into two distinct groups that composed of mainly G1 and G2 cells, respectively.

# Supplementary figures


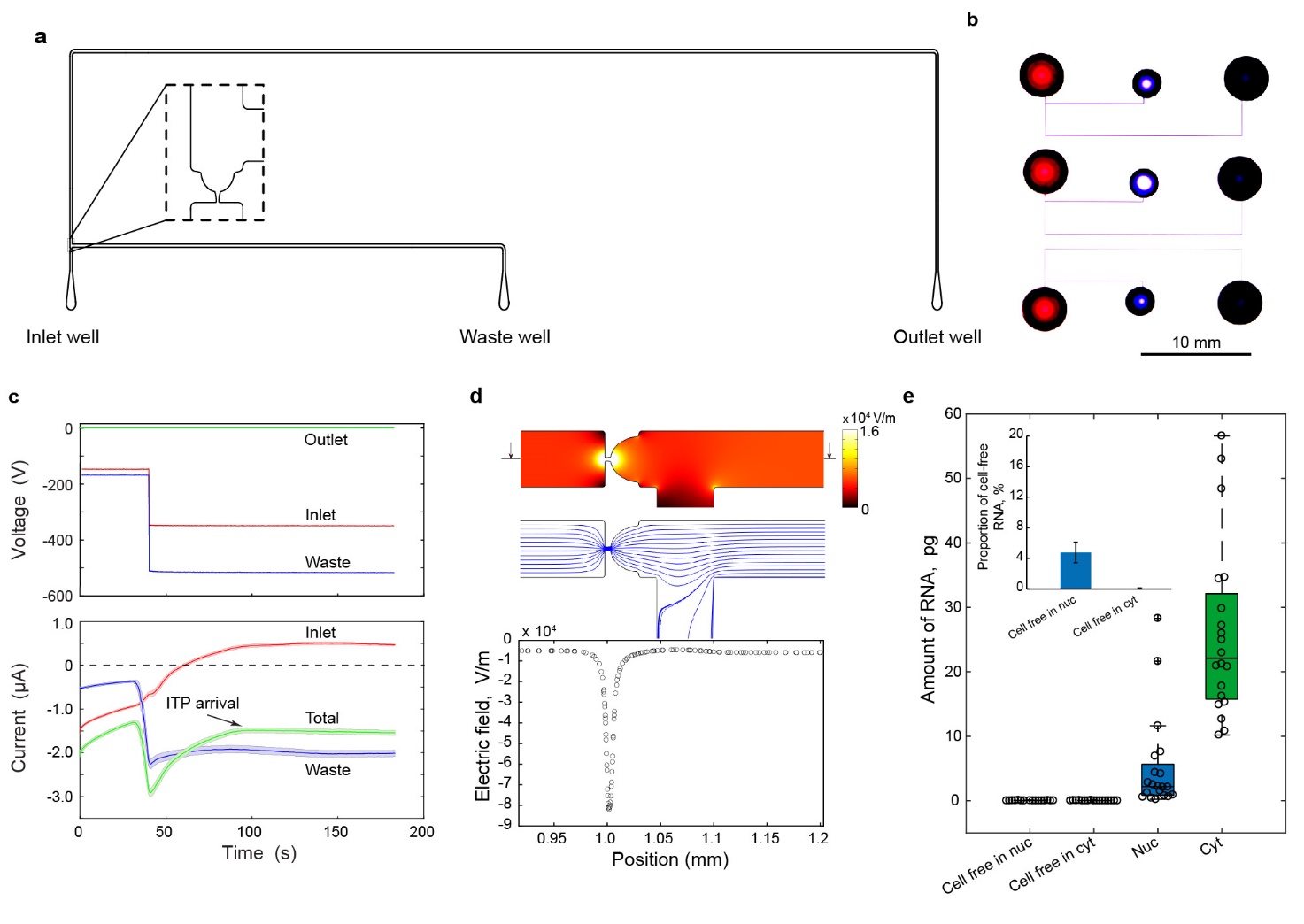


**Figure S1 Microfluidic system for SINC-seq protocol.** **a** Geometry of microfluidic channel with a hydrodynamic trap. **b** Photograph of the microfluidic chip consisting of three channels filled with food coloring. **c** DC voltage (upper panel) and ionic current behavior (lower panel) during the extraction protocol. The shade shows 95% confidence interval computed from 48 experiments. **d** Magnitude of electric field around the hydrodynamic trap (top panel) simulated with COMSOL, electric field lines concentrating at the trapping site (middle panel), and 15-fold electric field amplification at the trap (bottom panel). **e** Effect of cell-free RNA on extracted RNA sample. We performed experiments loading 1 μL of a supernatant of the cell suspension and quantified the amount of supernatant RNA with RT-PCR targeting *GAPDH*. We estimated the background RNA levels were 0.06±0.03(S.E.)% and 5.0±1.3(S.E.)% in cytRNA-seq and nucRNA-seq, respectively.


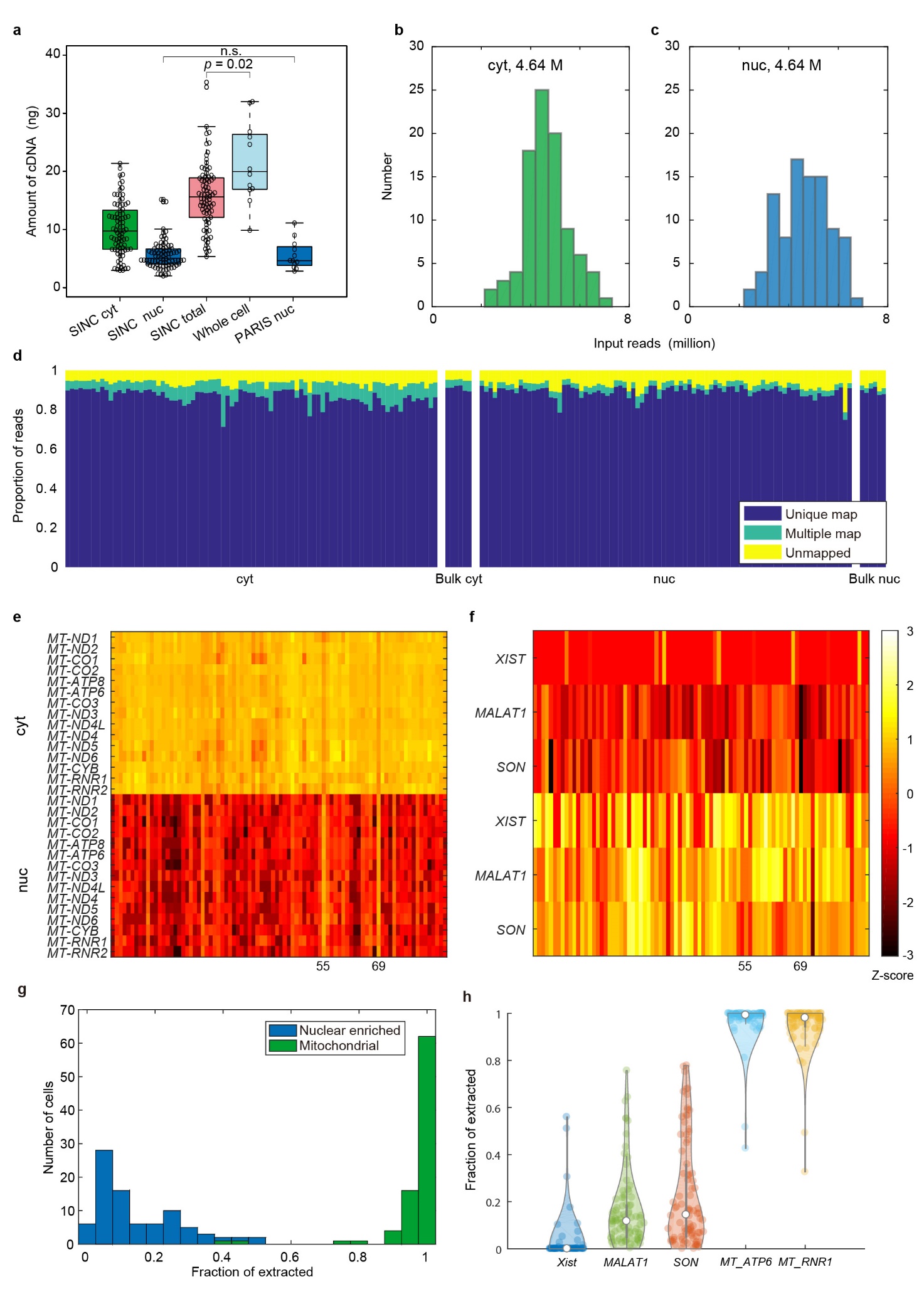


**Figure S2 Quality control data of SINC-seq.** **a** Yields of synthesized cDNA with cytRNA and nucRNA of SINC-seq. The total amount of cDNA was calculated by summing cDNA amounts of cytRNA and nucRNA to compare with the conventional single-cell protocol. Amount of cDNA synthesized with single nuclei isolated with PARIS Kit is also shown for comparison. **b, c** Input reads of cytRNA-seq and nucRNA-seq. **d** Proportion of reads of unmapped, multi-mapped, and uniquely mapped to the GRCh37.75 reference genome. **e,f** Heatmap of expression levels of mitochondrial RNA and nuclear-enriched RNA, respectively. **g** Fraction of extracted RNA to cytRNA estimated with mitochondrial RNAs and nuclear-enriched RNA, respectively. **h** Fraction of extracted estimated with individual genes.


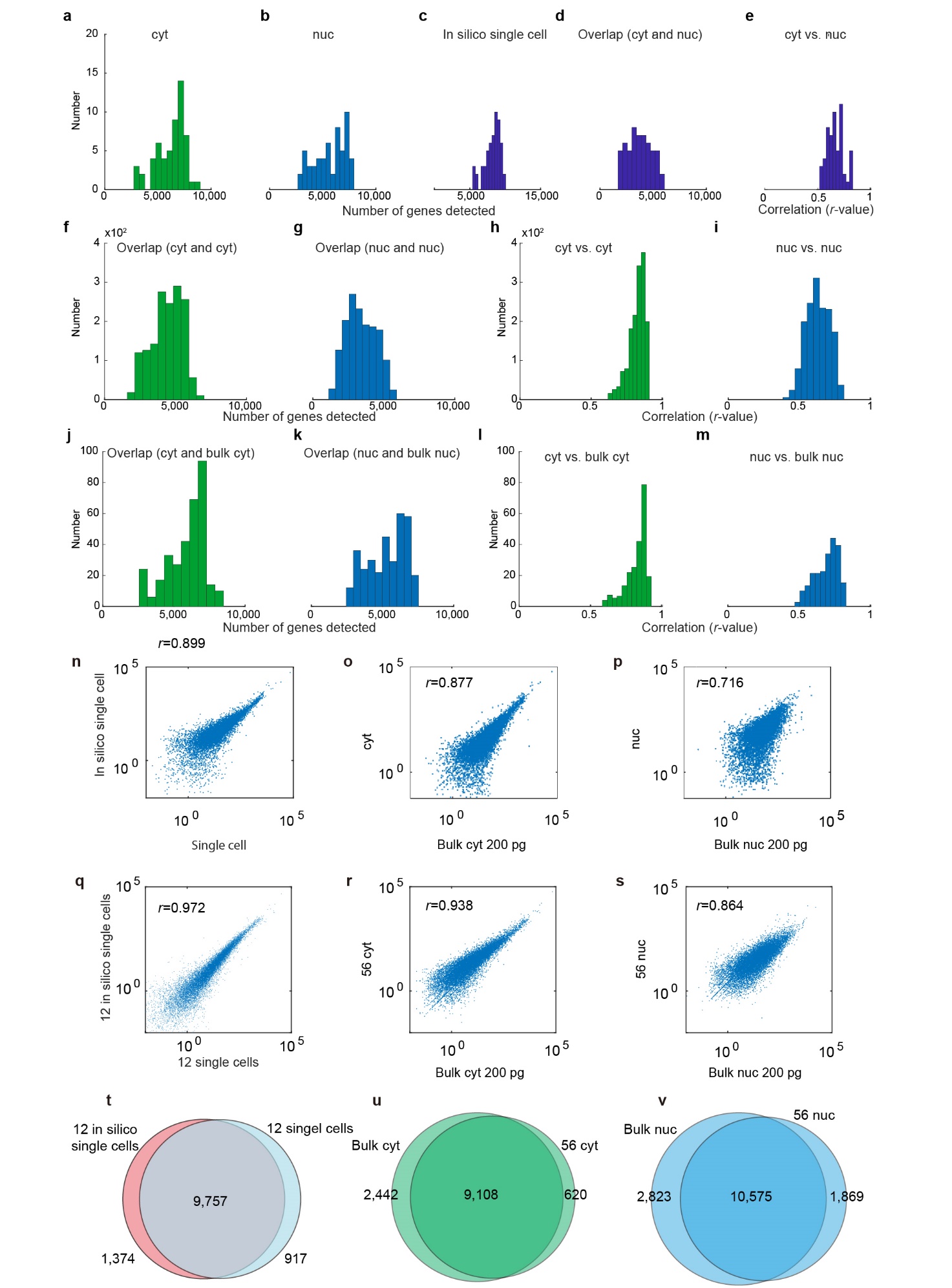


**Figure S3 Benchmark of SINC-seq with detection of genes. a-c** Numbers of detected genes with cytRNA, nucRNA, and in silico single cell data, respectively. **d** Numbers of genes overlapped in cytRNA and nucRNA. **e** Coefficient of correlation between cytRNA and nucRNA. **f, g** Numbers of repeatedly detected genes in a pair of cytRNA and in a pair of nucRNA, respectively. **h, i** Coefficients of correlation with a pair of cytRNAs and with a pair of nucRNAs. **j, k** Numbers of detected genes overlapped between cytRNA-seq and bulk cytRNA-seq, and between nucRNA-seq and bulk nucRNA-seq, respectively. **l, m** Coefficients of correlation between cytRNA-seq and bulk cytRNA-seq, and between nucRNA-seq and bulk nucRNA-seq, respectively. **n** Correlation of gene expression between in silico single-cell data and scRNA-seq; **o** cytRNA-seq and bulk cytRNA-seq; **p** nucRNA-seq and bulk nucRNA-seq. **q** Correlation of gene expression between 12 in silico single-cell data and 12 scRNA-seq; **r** 56 cytRNA-seq and bulk cytRNA-seq; **s** 56 nucRNA-seq and bulk nucRNA-seq. **t** Venn diagrams of detected genes with in silico normalized data for 12 single cells and conventional 12 scRNA-seq; **u** with 56 cytRNA-seq and bulk cyt RNA-seq; **v** with 56 nucRNA-seq and bulk nucRNA-seq.


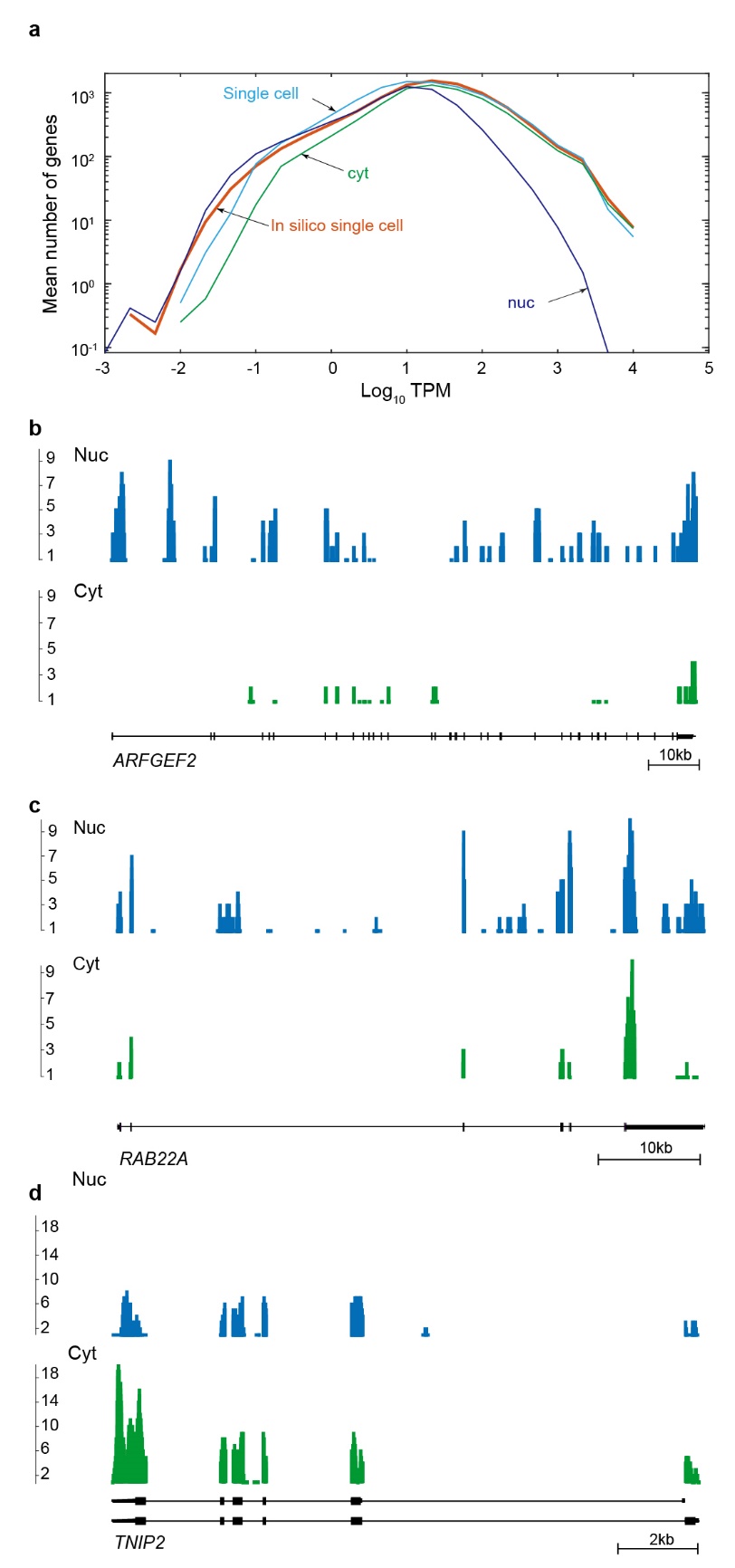


**Figure S4 In silico single cell data shows a wider dynamic range of detected genes integrating cytRNA-seq and nucRNA-seq. a** Gene density plot of nucRNA-seq, cytRNA-seq, scRNA-seq and in silico scRNA-seq. **b-d** Coverages of low abundant RNAs, *ARFGEF2*, *RAB22A*, and *TNIP2*, detected in nucRNA with TPM~0.01.


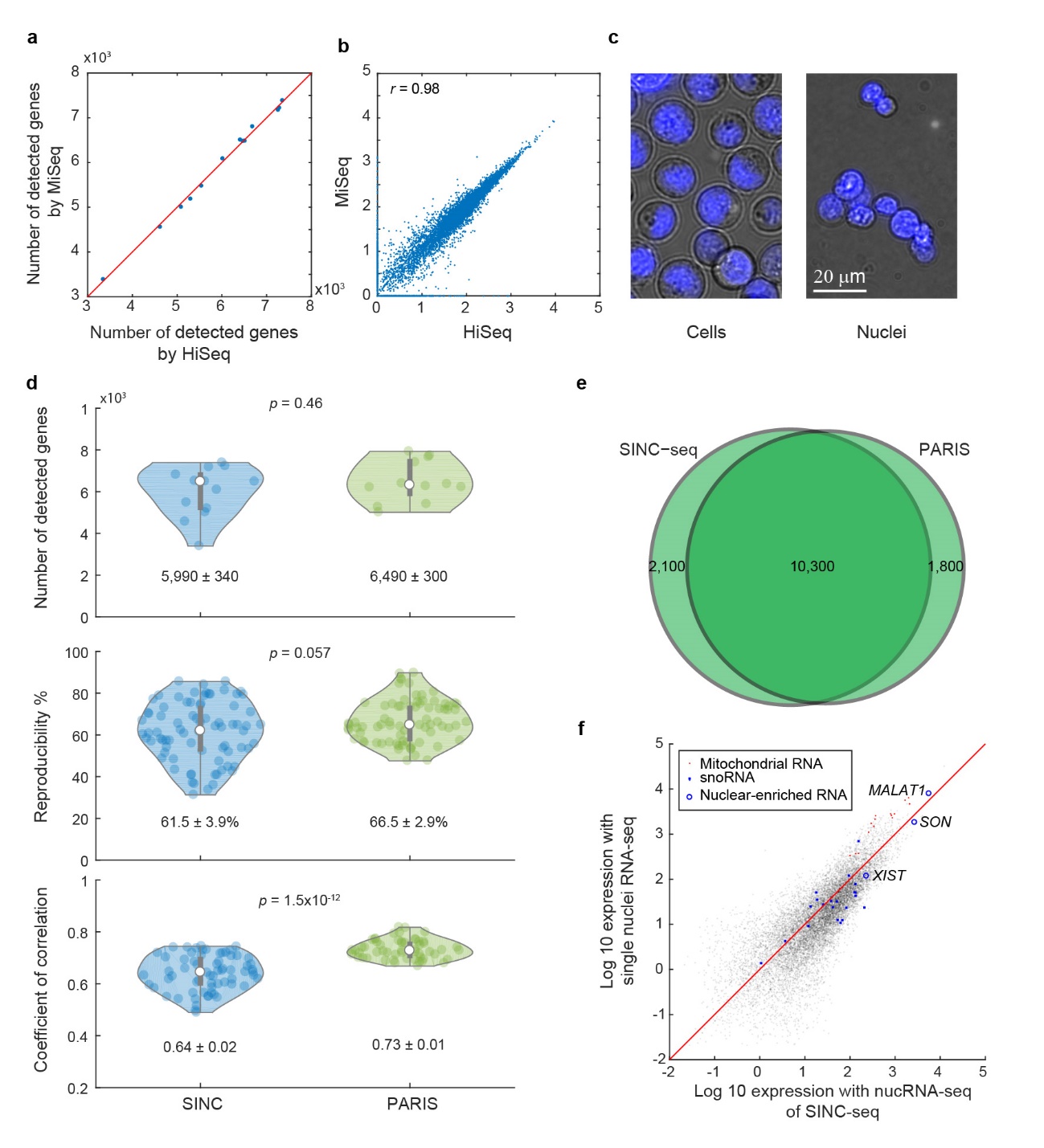


**Figure S5 Comparison of nucRNA-seq of SINC-seq versus single nuclei RNA-seq. a** Comparison of number of detected genes with nucRNA-seq of SINC-seq using HiSeq2500 and MiSeq. **b** Comparison of gene expression patterns obtained with HiSeq and MiSeq. **c** K562 cells stained with Hoechst and nuclei prepared by the off-the-shelf kit. **d** Comparison of nucRNA-seq of SINC-seq versus single nuclei RNA-seq with number of detected genes, reproducibility of gene detection, and coefficient of correlation. **e** Venn diagram of number of detected genes for 12 nucRNA-seq and 12 single nuclei RNA-seq. **f** Comparison of gene expression pattern between 12 nucRNA-seq and 12 single nuclei RNA-seq.


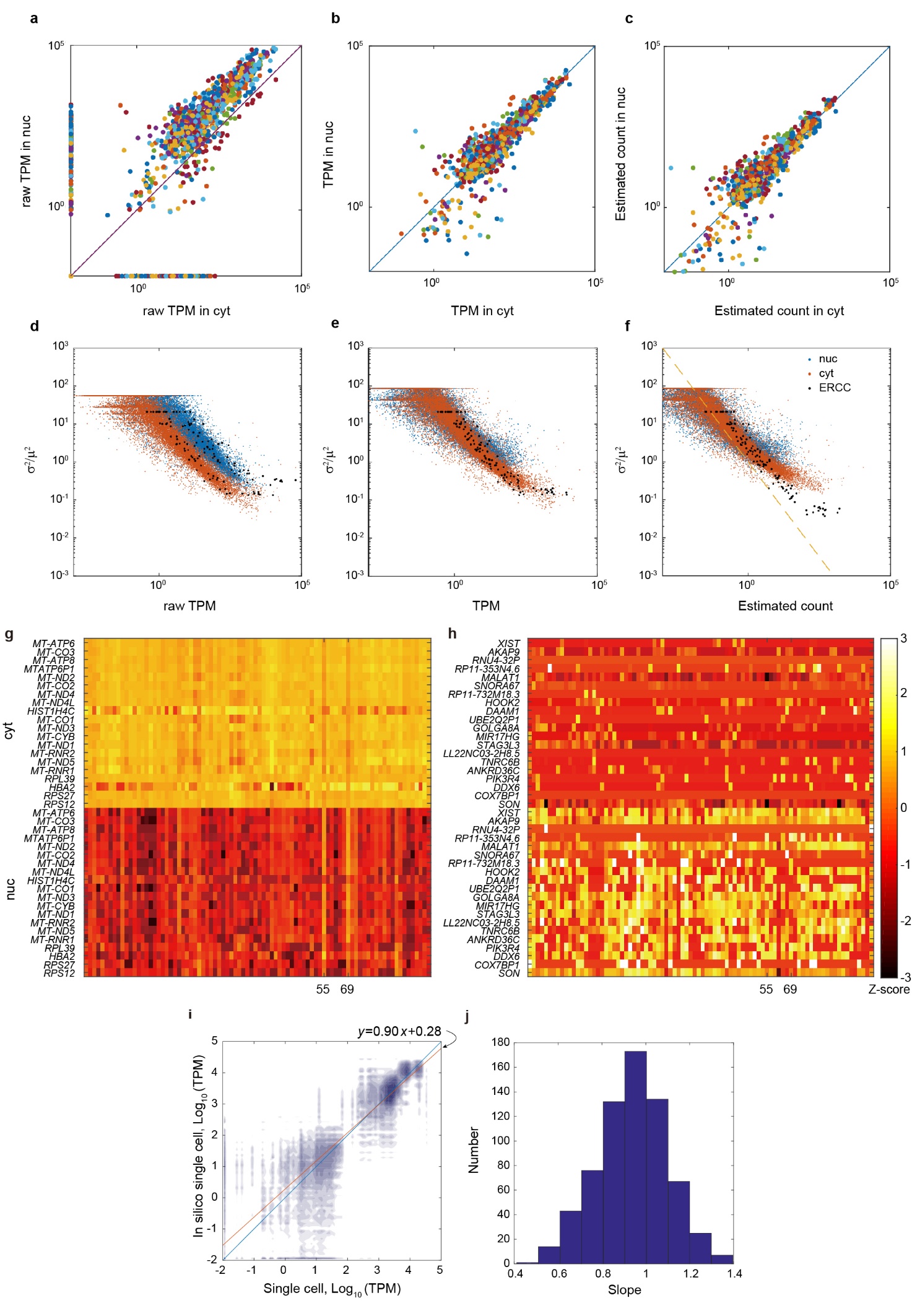


**Figure S6 Scaling cytRNA-seq and nucRNA-seq for generating in silico single-cell data. a-c** ERCC expression comparing cytRNA-seq versus nucRNA-seq with raw TPM, TPM, and estimated counts, respectively. **d-f** CV^2^ versus mean plots with raw TPM, TPM and estimated counts, respectively. **g, h** Fractionation stringency assessed with top 20 genes enriched in cytRNA and nucRNA, respectively. **i** Comparison of expression patterns of the top 20 genes enriched in cytRNA and nucRNA between in silico single-cell data and scRNA-seq. **j** Statistics of the slope comparing in silico single cell data versus scRNA-seq with the expression level of the top 20 localized genes.


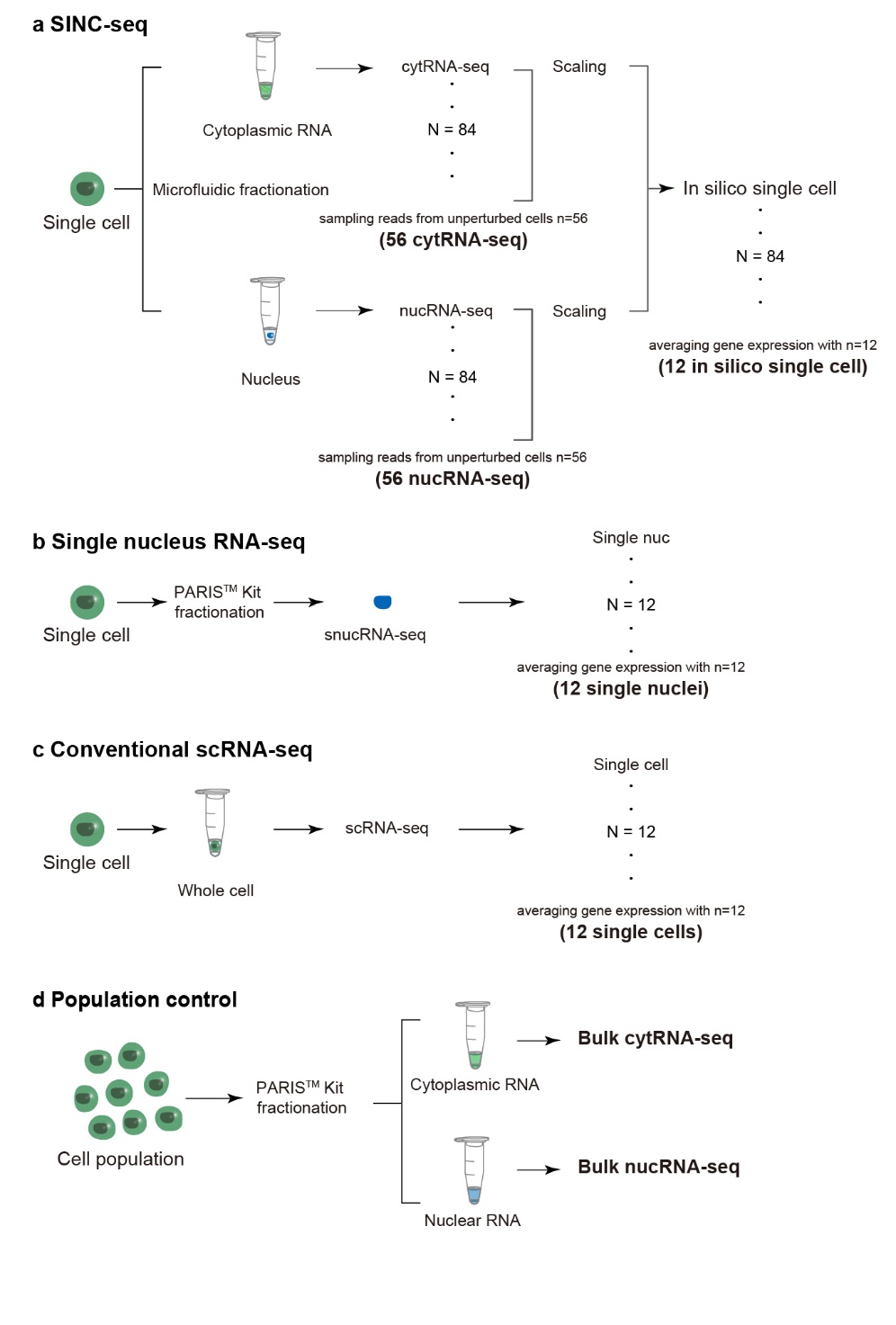


**Figure S7 Overview of RNA-seq samples.** **a** SINC-seq constructs cytRNA-seq and nucRNA-seq per cell with cytoplasmic RNA and a nucleus, respectively. 56 cytRNA-seq and 56 nucRNA-seq data were, respectively, created by randomly sampling reads from 56 of cytRNA-seq and 56 of nucRNA-seq. In silico single cell data were created by scaling and integrating cytRNA-seq and nucRNA-seq from the same single cell. The 12 in silico single cell data was created by averaging 12 of randomly sampled in silico single cell data sets. **b** Single nucleus RNA-seq, for which nuclei were prepared with PARIS Kit. The 12 single nuclei RNA-seq was created by averaging the 12 of single nuclei RNA-seq. **c** Conventional scRNA-seq. 12 single cell RNA-seq was created by averaging the 12 of scRNA-seq. **c** Population controls of bulk cytRNA-seq and bulk nucRNA-seq were prepared with PARIS Kit, which fractionates cytoplasmic RNA and nuclear RNA with a population of cells, followed by Smart-seq2 protocol with 200 pg RNA and 15 PCR cycles.


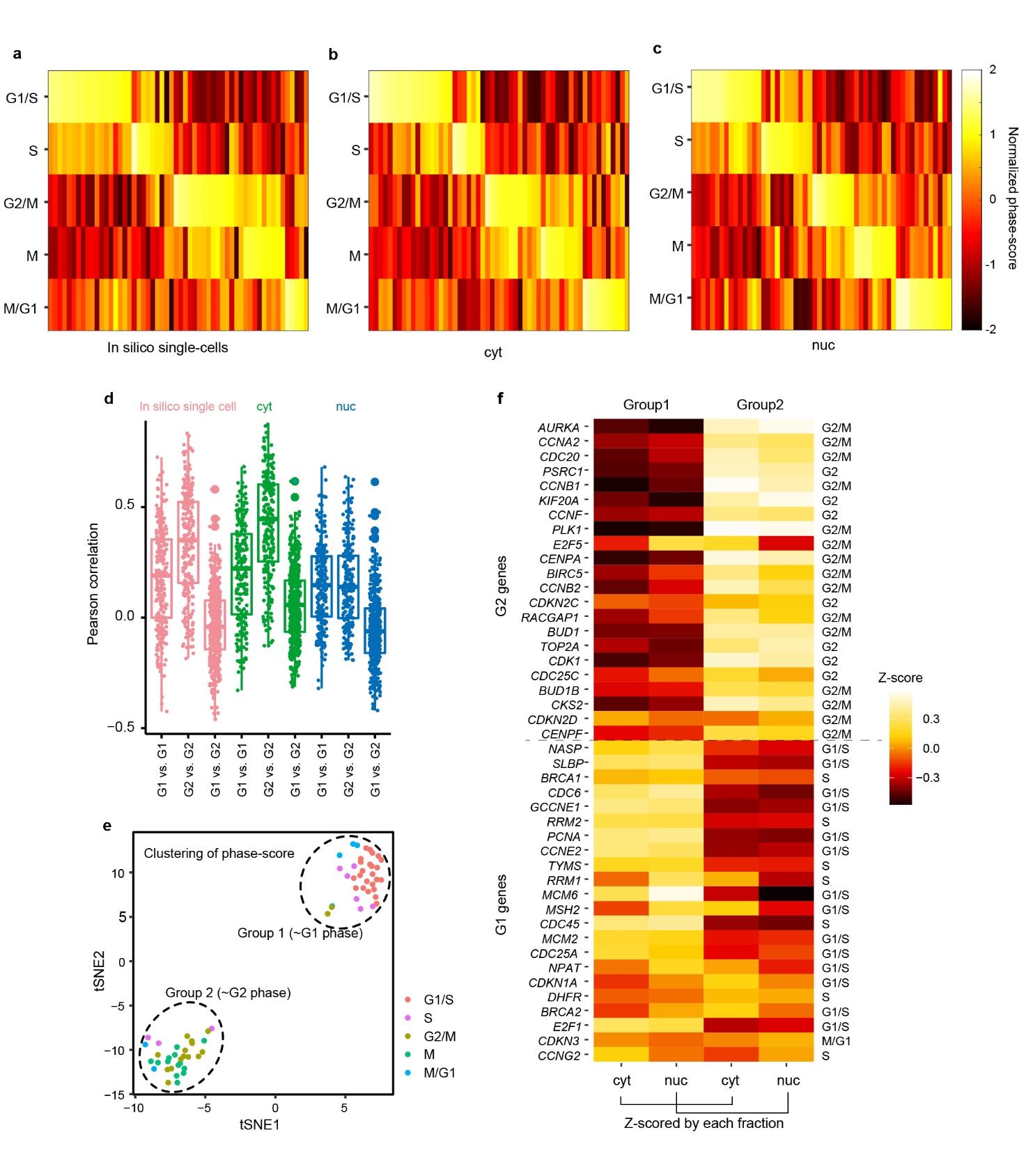


**Figure S8 Cell-cycle phase of K562 cells analyzed by SINC-seq. a-c** Normalized phase scores of in silico single cell data, cytRNA-seq, and nucRNA-seq, respectively. **d** Pearson correlations shown in Fig. 2 d-f. **e** tSNE with normalized phase scores (Fig. S8a) segregates cells into G1 and G2 groups. **f** Z-score calculated with individual fraction indicates relative expression of each gene among G1 and G2 groups.


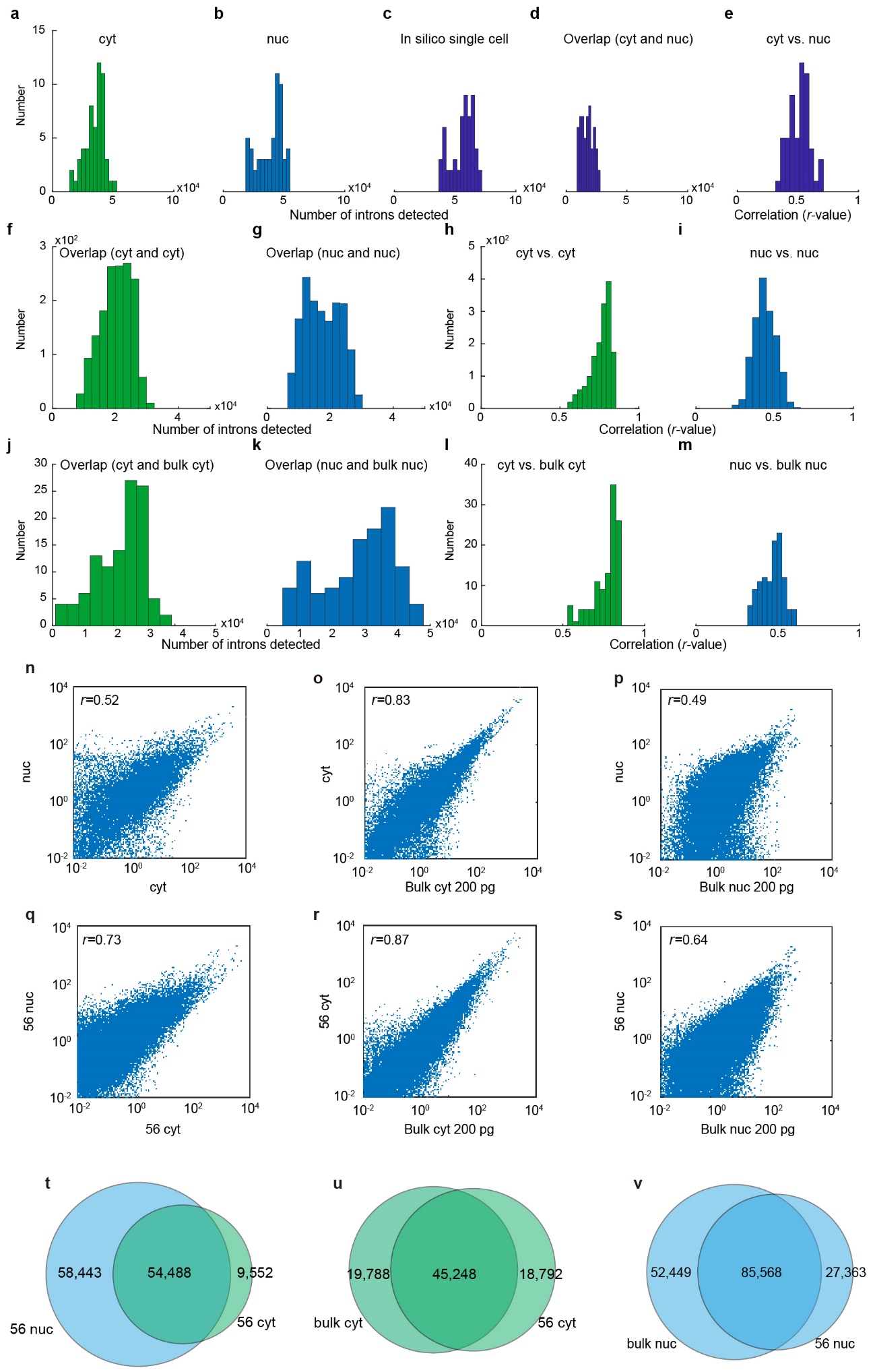


**Figure S9 Benchmark of SINC-seq with detection of introns.** **a-c** Numbers of detected introns with cytRNA, nucRNA, and in silico single cell data, respectively. **d** Number of detected introns overlapped in cytRNA and nucRNA. **e** Coefficient of correlation with intron abundance between cytRNA and nucRNA. **f, g** Numbers of detected introns in a pair of cytRNAs and in a pair of nucRNAs, respectively. **h, i** Coefficients of correlation with a pair of cytRNAs and a pair of nucRNAs, respectively. **j, k** Numbers of detected introns overlapped cytRNA-seq and bulk cytRNA-seq, and nucRNA-seq and bulk nucRNA-seq, respectively. **l, m** Coefficients of correlation between cytRNA-seq and bulk cytRNA-seq, and between nucRNA-seq and bulk nucRNA-seq, respectively. **n** Correlation of intron abundance between cytRNA-seq and nucRNA-seq; **o** cytRNA-seq and bulk cytRNA-seq; **p** nucRNA-seq and bulk nucRNA-seq. **q** Correlation of intron abundance between 56 cytRNA-seq and 56 nucRNA-seq; **r** 56 cytRNA-seq and bulk cytRNA-seq; **s** 56 nucRNA-seq and bulk nucRNA-seq. **t** Venn diagrams of detected introns with 12 cytRNA-seq and 12 nucRNA-seq; **u** with 56 cytRNA-seq and bulk cytRNA-seq; **v** with 56 nucRNA-seq and bulk nucRNA-seq.


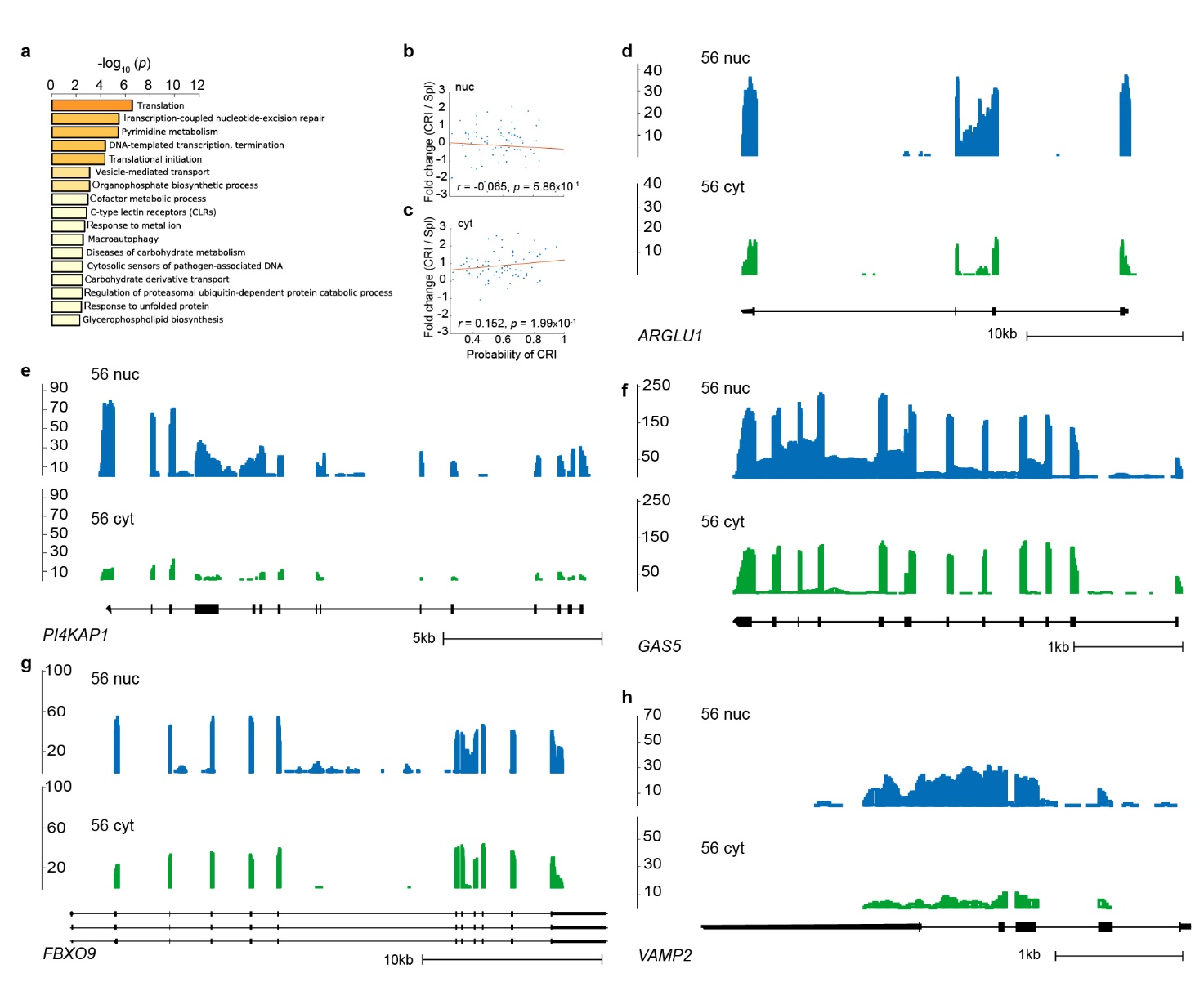


**Figure S10 NRI and CRI have different enriched functions. a** Gene ontology analysis with CRI. **b, c** Correlation analysis between the probability of CRI and the fold change of gene expression among cells with CRI and without CRI (Spl: spliced) in nucRNA and cytRNA, respectively. Coverages of **d** *ARGLU1*, **e** *PI4KAP1*, **f** *GAS5* (*SNHG2*), **g** *FBXO9*, and **h** *VAMP2* genes.


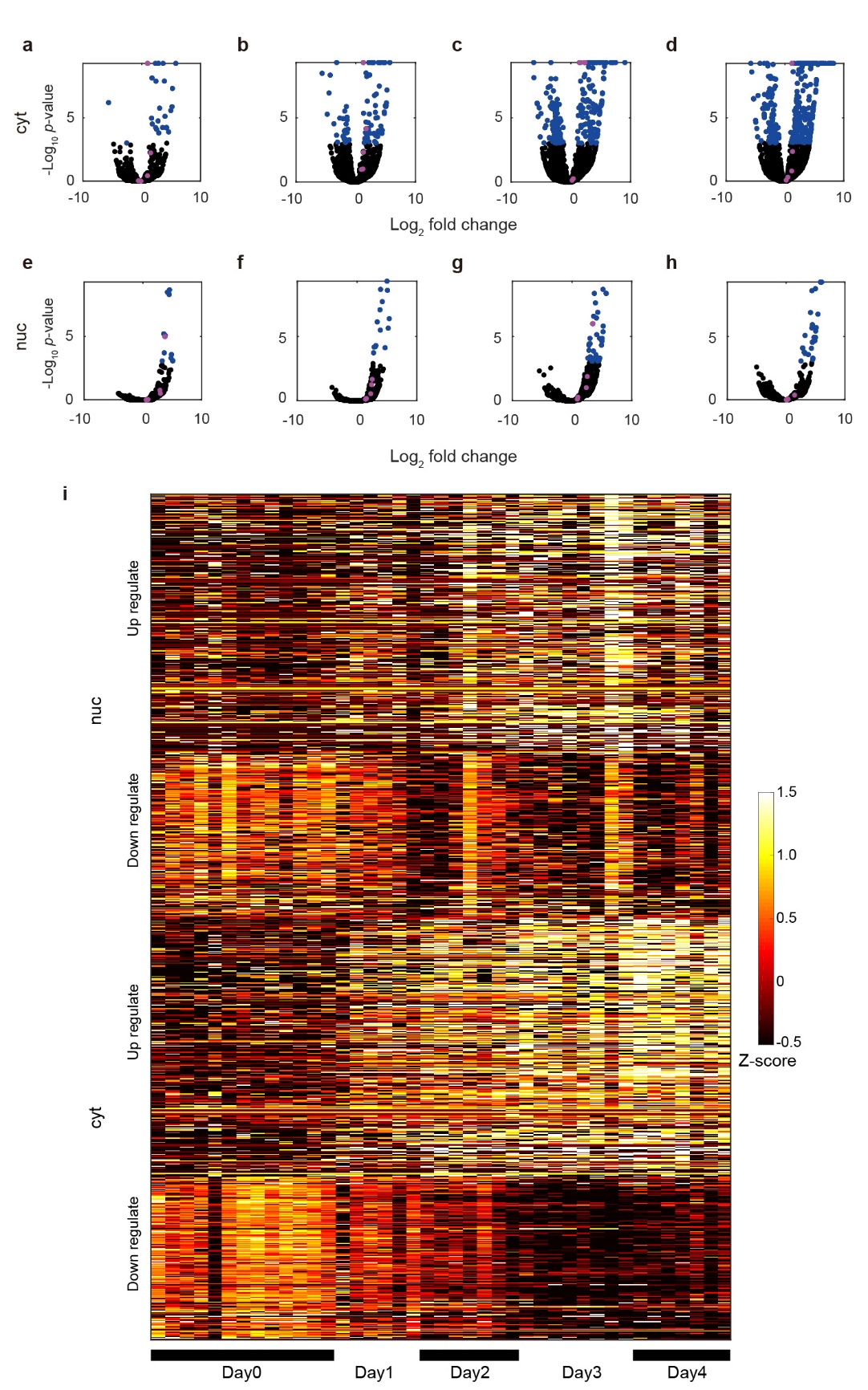


**Figure S11 Differentially expressed genes along sodium butyrate-induced K562 differentiation.** **a-d** Differential expression with cytRNA of day 0 vs. day 1, day 0 vs. day 2, day 0 vs. day 3, and day 0 vs. day 4, respectively. **e-h** Differential expression with nucRNA of day 0 vs. day 1, day 0 vs. day 2, day 0 vs. day 3, and day 0 vs. day4, respectively. We identified DEG using “nbintest” and “mafdr” of MATLAB functions. Blue, genes with *p* values less than 0.001 and absolute log2 fold changes greater than unity. Pink, *GATA1*, *HBG1*, *HBG2*, *GYPA* and *TFRC* genes. **i** Heatmap showing up- and down-regulations of DEG in cytRNA and nucRNA.


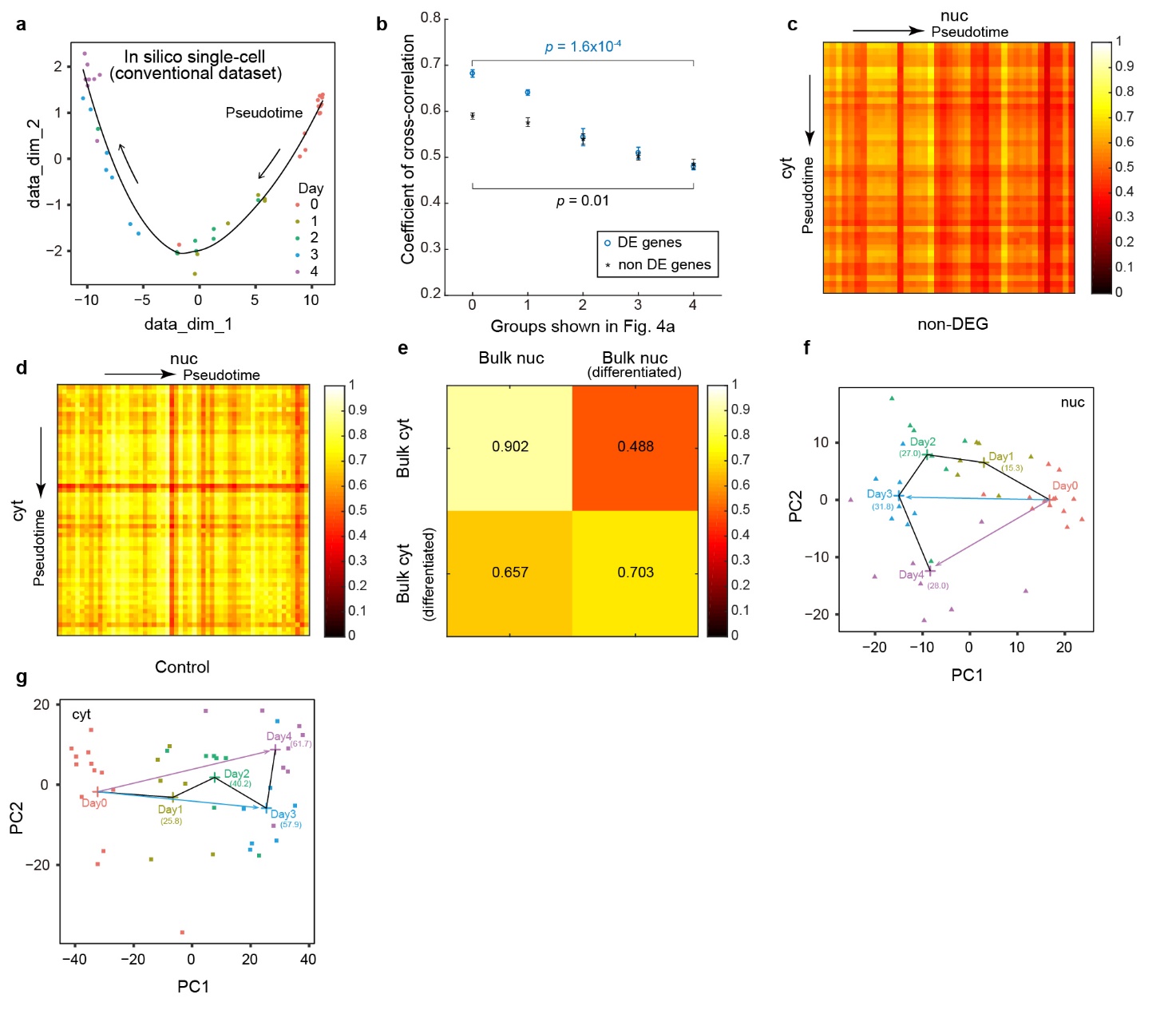


**Figure S12 Correlation dynamics under sodium butyrate differentiation of K562 cells.** **a** Pseudotime computed with Monocle (version 2.4.0) [7, 8] using DEG expression of in silico single-cell data. **b** Coefficient of cross-correlation between cytRNA and nucRNA along the differentiation of K562 cells. **c** Cross-correlation between cytRNA and nucRNA computed with non-DEG of differentiating K562 cells. **d** Cross-correlation of cytRNA and nucRNA computed with DEG of non-differentiating K562 cells along pseudotime. **e** Cross-correlation of bulk cytRNA-seq and bulk nucRNA-seq computed with DEG of differentiating K562 cells. **f, g** PCA on nucRNA-seq and cytRNA-seq data of differentiating K562 cells, respectively. Cross-points in these panels indicate the center of mass of each cluster.

# Reference

1. Hutchinson, J.N., et al., *A screen for nuclear transcripts identifies two linked noncoding RNAs associated with SC35 splicing domains.* BMC Genomics, 2007. **8**: 39.

2. Sharma, A., et al., *Son Is Essential for Nuclear Speckle Organization and Cell Cycle Progression.* Molecular Biology of the Cell, 2010. **21**(4): p. 650-663.

3. Yang, F., et al., *MALAT-1 interacts with hnRNP C in cell cycle regulation.* Febs Letters, 2013. **587**(19): p. 3175-3181.

4. Wu, A.R., et al., *Quantitative assessment of single-cell RNA-sequencing methods.* Nat Methods, 2014. **11**(1): p. 41-46.

5. Whitfield, M.L., et al., *Identification of genes periodically expressed in the human cell cycle and their expression in tumors.* Molecular Biology of the Cell, 2002. **13**(6): p. 1977-2000.

6. Macosko, E.Z., et al., *Highly Parallel Genome-wide Expression Profiling of Individual Cells Using Nanoliter Droplets.* Cell, 2015. **161**(5): p. 1202-1214.

7. Qiu, X.J., et al., *Single-cell mRNA quantification and differential analysis with Census.* Nature Methods, 2017. **14**(3): p. 309-315.

8. Trapnell, C., et al., *The dynamics and regulators of cell fate decisions are revealed by pseudotemporal ordering of single cells.* Nature Biotechnology, 2014. **32**(4): p. 381-386.
